# Clamping, bending, and twisting inter-domain motions in the misfold-recognizing portion of UDP-glucose: Glycoprotein glucosyltransferase

**DOI:** 10.1016/j.str.2020.11.017

**Published:** 2021-04-01

**Authors:** Carlos P. Modenutti, Juan I. Blanco Capurro, Roberta Ibba, Dominic S. Alonzi, Mauro N. Song, Snežana Vasiljević, Abhinav Kumar, Anu V. Chandran, Gabor Tax, Lucia Marti, Johan C. Hill, Andrea Lia, Mario Hensen, Thomas Waksman, Jonathan Rushton, Simone Rubichi, Angelo Santino, Marcelo A. Martí, Nicole Zitzmann, Pietro Roversi

**Affiliations:** 1 Departamento de Química Biológica, Facultad de Ciencias Exactas y Naturales, Universidad de Buenos Aires, Ciudad Universitaria, Pab. II (CE1428EHA), Buenos Aires, Argentina; 2 Instituto de Química Biológica de la Facultad de Ciencias Exactas y Naturales (IQUIBICEN) CONICET. Ciudad Universitaria, Pab. II (CE1428EHA), Buenos Aires, Argentina; 3 Oxford Glycobiology Institute, Department of Biochemistry, University of Oxford, Oxford OX1 3QU, UK; 4 Dipartimento di Chimica e Farmacia, Università degli Studi di Sassari, Via Muroni 23A, 07100 Sassari, SS, Italy; 5 Leicester Institute of Structural and Chemical Biology, Department of Molecular and Cell Biology, University of Leicester, Henry Wellcome Building, Lancaster Road, Leicester, LE1 7RH,, UK; 6 Institute of Sciences of Food Production, C.N.R. Unit of Lecce, via Monteroni, 73100 Lecce, Italy

**Keywords:** UGGT, glycoprotein folding, negative-stain EM, molecular dynamics, Parodi limit, misfolding, misfold sensing, X-ray diffraction, re-glucosylation, GT24 domain

## Abstract

UDP-glucose:glycoprotein glucosyltransferase (UGGT) flags misfolded glycoproteins for ER retention. We report crystal structures of full-length *Chaetomium thermophilum* UGGT (*Ct*UGGT), two *Ct*UGGT double-cysteine mutants, and its TRXL2 domain truncation (*Ct*UGGT-ΔTRXL2). *Ct*UGGT molecular dynamics (MD) simulations capture extended conformations and reveal clamping, bending, and twisting inter-domain movements. We name “Parodi limit” the maximum distance on the same glycoprotein between a site of misfolding and an N-linked glycan that can be reglucosylated by monomeric UGGT *in vitro*, in response to recognition of misfold at that site. Based on the MD simulations, we estimate the Parodi limit as around 70–80 Å. Frequency distributions of distances between glycoprotein residues and their closest N-linked glycosylation sites in glycoprotein crystal structures suggests relevance of the Parodi limit to UGGT activity *in vivo*. Our data support a “one-size-fits-all adjustable spanner” UGGT substrate recognition model, with an essential role for the UGGT TRXL2 domain.

## Highlights

• UGGT MD simulations widen the conformational range observed in crystal structures
• The UGGT TRXL2 domain is essential for enzymatic activity
• A misfold site is closer than the Parodi limit to a glycan in UGGT clients *in vitro*
• N-Glycan distributions suggest evolution optimizes glycoprotein surface coverage

## Graphical abstract

**Figure:**
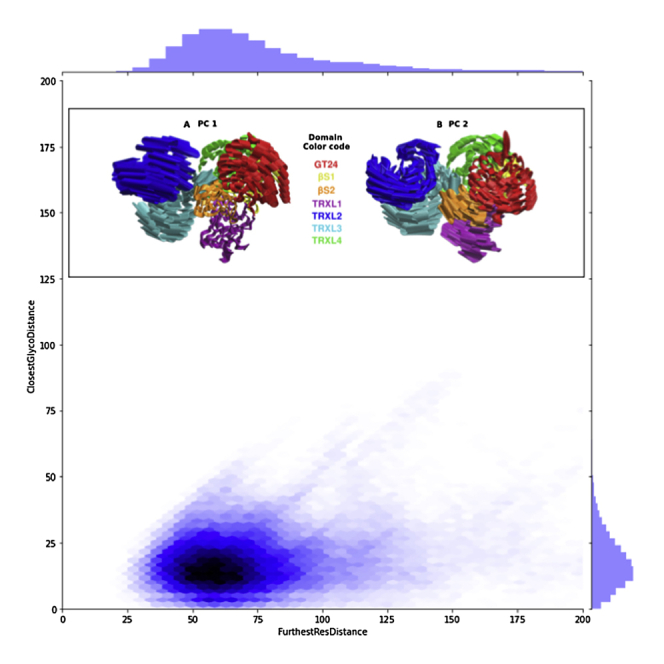

## Teaser

Modenutti et al. carry out molecular dynamics simulations of UGGT, the enzyme surveying correct folding of glycoproteins, and propose a “one-size-fits all adjustable spanner” UGGT:substrate recognition model. The UGGT TRXL2 domain is essential for its function. The size of UGGT likely dictates restraints on the evolution of N-linked glycosylation sites.

## Introduction

A wonderfully efficient protein-folding machinery in the ER of eukaryotic cells ensures that only correctly folded glycoproteins can exit the ER, proceed to the Golgi, and from there continue along the secretory pathway toward their cellular or extracellular destinations (Vincenz-Donnelly and Hipp, 2017). The stringency of this ER quality control (ERQC) system is of great advantage to healthy cells. It allows time for complex glycoproteins to fold in the ER and prevents premature secretion of incompletely folded species. In the background of a misfold-inducing missense mutation in a secreted glycoprotein gene, the resulting misfolded glycoprotein is either retained in the ER by ERQC or degraded by the ER-associated degradation (ERAD) machinery (Amara et al., 1992). ERQC-mediated ER retention and ERAD degradation of glycoprotein mutants bear particularly unfortunate consequences when the mutation induces a minor folding defect but does not abrogate the function of the glycoprotein (“responsive mutant”). In these cases ERQC/ERAD cause disease by blocking the secretion of the glycoprotein mutant, even though its residual activity would be beneficial to the organism (see for example Parodi et al., 2014).

Central to ERQC is the ER-resident 170-kDa enzyme UDP-glucose:glycoprotein glucosyltransferase (UGGT). The enzyme selectively reglucosylates a misfolded glycoprotein on one of its N-glycans and promotes its association with the ER lectins calnexin and calreticulin, thus mediating its ER retention. More than 25 years after the discovery of UGGT (Parodi, 2007; Parodi et al., 2014), recent structural and functional work has uncovered the protein's multi-domain architecture and provided preliminary evidence of its inter-domain conformational flexibility (Calles-Garcia et al., 2017; Roversi et al., 2017; Satoh et al., 2017). Here, we use molecular dynamics (MD) to further characterize UGGT's inter-domain flexibility and present recently obtained *Ct*UGGT crystal structures and activity data. We define and give a numerical estimate of the “Parodi limit,” the maximum distance between a site of misfolding and an N-linked glycan that can be reglucosylated by monomeric UGGT on the same glycoprotein *in vitro* in response to recognition of misfold at that site. The MD trajectories are discussed in the light of all the available structural and functional data, supporting a one-size-fits-all model of UGGT promiscuity, with an essential role for the UGGT TRXL2 domain.

## Results

### The *Ct*UGGT_Kif_ crystal structure adds to the landscape sampled by previously observed UGGT conformations

**Figure 1:**
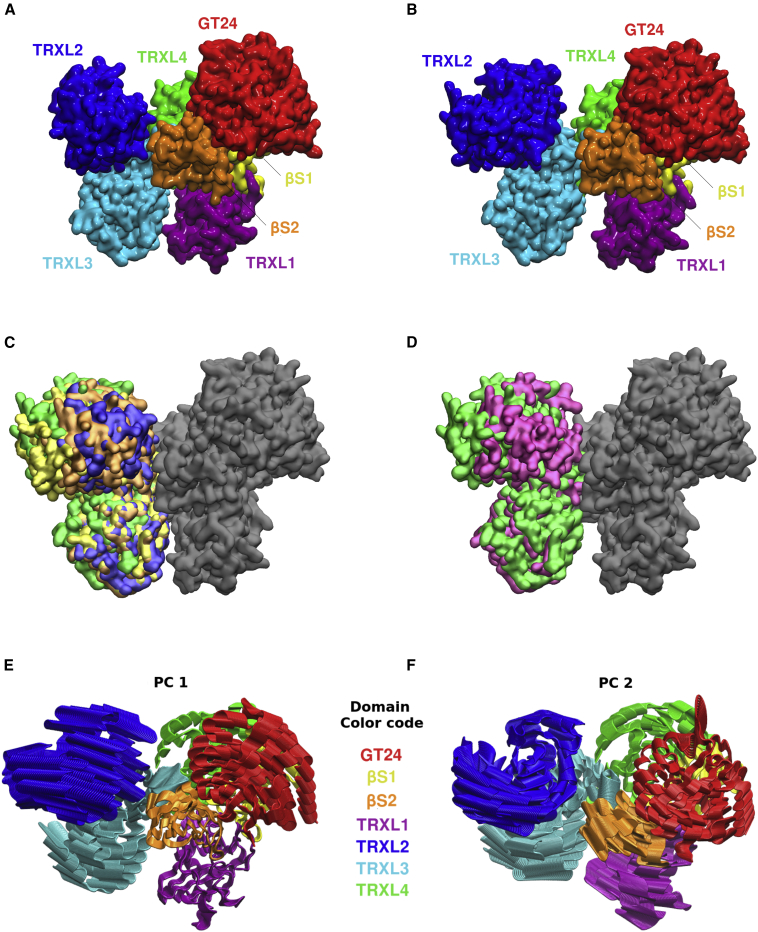
Crystal structures of *Ct*UGGT and the first two principal components of the joint MD simulations (A and B) Structural comparison between (A) *Ct*UGGT in “closed” (PDB: 5N2J) and (B) “open” (PDB: 5MZO) conformations, colored domain by domain: TRXL1 (residues 45–220), magenta; TRXL2 (residues 414–656), blue; TRXL3 (residues 667–880), cyan; TRXL4 (residues 275–410; 897–950), green; βS1 (residues 28–36; 225–242; 957–1037), yellow; βS2 (residues 1,039–1,149), orange; GT24 (residues 1,197–1,475), red. (C) Superimpositions of all four *Ct*UGGT X-ray structures available prior to this publication; domains colored in gray (GT24, βS1, βS2, and TRXL4) represent the relatively rigid portion of the molecule (RMSD_Cα_ less than 0.750 Å), which was used to align the structures. TRXL2 and TRXL3 domains are colored as follows: purple, “closed” conformation (PDB: 5N2J); orange, D611C-G1050C mutant also known as “closed-like” conformation (PDB: 5NV4); yellow, “intermediate” conformation (PDB: 5MU1); green, “open” conformation (PDB: 5MZO). (D) Superimposition of the “open” conformation (TRXL2 and TRXL3 domains in green) with the recently reported “new-intermediate” *Ct*UGGT_Kif_ conformation (TRXL2 and TRXL3 domains in magenta) (PDB: 6TRF). (E and F) The first two principal components (PCs) of the joint MDs. Domains colored as in (A) and (B). Figures made in VMD (Cross et al., 2009).

**Figure 2:**
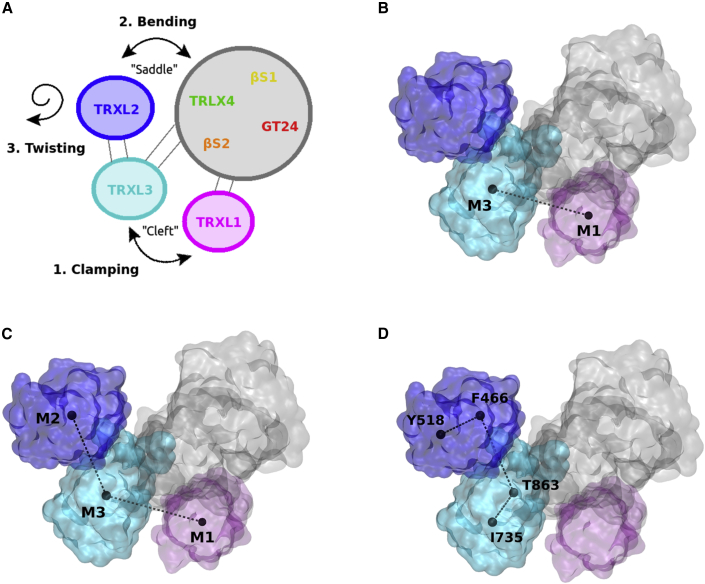
Main UGGT motions and conformational coordinates (A) Simplified representation of *Ct*UGGT overall movements. “Clamping” movement between domains TRXL3 and TRXL1; “bending” movement between TRXL2 and the core comprising domains GT24-βS1-βS2-TRXL4; “twisting” movement of TRXL2 with respect to TRXL3. The gray area represents the strong structural inter-domain orientation invariance of the TRXL4-βS1-βS2-GT24 domains. (B–D) Conformational coordinates (CCs) for describing *Ct*UGGT conformational states: TRXL1 (magenta), TRXL2 (blue), and TRXL3 (cyan). (B) Along “CC1,” the “clamping” coordinate measures the openness of the cleft between the TRXL1 and TRXL3 domains. (C) Along “CC2,” the “bending” coordinate measures the distance between the TRXL2 and GT24 domains across the central saddle. (D) Along “CC3,” the “twisting” coordinate changes with the relative orientation of the TRXL2 and TRXL3 domains. (B), (C), and (D) made in VMD (Cross et al., 2009).

The full-length *Chaetomium thermophilum* UGGT (*Ct*UGGT) crystal structures revealed four DsbA-like domains (TRXL1–4) arranged in a long arc, terminating in two β sandwiches (βS1 and βS2) tightly clasping the glucosyltransferase family 24 (GT24) domain (Figures 1A and 1B) (Roversi et al., 2017). These UGGT domains are labeled as thioredoxin-like (TRXL) but strictly speaking, the UGGT TRXL2-4 domains belong to a modified version of the thioredoxin fold, the DsbA-like fold: βαβ-αααα-αββα (Pfam DSBA family PF01323). This fold has an extra four-helical subdomain capping one side of a thioredoxin domain βαβ-αββα (Kozlov and Gehring, 2020). The UGGT-TRXL1 domain has a slightly altered and unique topology, with the four-helical subdomain inserted before the thioredoxin one: αααα-βαβ-αββα (Kryshtafovych et al., 2018). The wild-type protein crystallized in three different conformations, called “closed” (PDB: 5N2J, Figure 1A, and gray with purple TRXL2 and TRXL3 domains in Figure 1C), “open” (PDB: 5MZO, Figure 1B and gray with green TRXL2 and TRXL3 domains in Figure 1C), and “intermediate” (PDB: 5MU1, gray with yellow TRXL2 and TRXL3 domains in Figure 1C) (Roversi et al., 2017). Additionally, the mutant *Ct*UGGT^D611C/G1050C^, engineered to form an extra disulfide bridge between the TRXL2 and βS2 domains, was trapped in a “closed-like” conformation (PDB: 5NV4, gray with orange TRXL2 and TRXL3 domains in Figure 1C). Those four *Ct*UGGT structures mainly differ in the spatial organization of domains TRXL2 and TRXL3 (respectively blue and cyan in Figures 1A, 1B, and 2, and Video S1). The TRXL2 domain is rotated by different amounts with respect to the rest of the protein and adopts different degrees of proximity to it. The TRXL3 domain instead appears in the same relative conformation in all structures, except for the “open” one, in which the TRXL3 and TRXL1 domains move apart, leading to the opening of a cleft between them (Figures 1B and 2A).

Video S1: *Ct*UGGT structures, related to Figure 1 Five crystal structures of *Ct*UGGT, in surface representation, with domains TRXL2 and TRXL3 in color, and the rest of the protein in gray

We describe here a fifth full-length *Ct*UGGT structure (hereafter *Ct*UGGT_Kif_, PDB: 6TRF), obtained from recombinant protein purified from mammalian cells treated with the mannosidase inhibitor kifunensine. We used this mannosidase inhibitor to prevent elaboration of N-linked glycans along the secretory pathway and ensure homogeneous high-mannose glycosylation, in the hope of obtaining better diffracting crystals. Despite carrying mostly high-mannose glycans as expected (see http://doi.org/10.5281/zenodo.3608191), *Ct*UGGT_kif_ yielded a crystal which diffracted only to 4.1 Å. Overall the thermal motion is rather high (<B> = 270 Å^2^, see Table S3). This is likely due to loose crystal packing. Alternatively, kifunensine has inhibited ERAD mannosidases and our crystal may have grown from a mixture of molecules bearing minor folding defects: the high B factors in this case would be modeling static disorder. In addition to the three regions that are usually disordered in *Ct*UGGT crystal structures (namely the TRXL4 loop, residues 246–276; the flexible linker between BS2 and the GT24 domain, residues 1,153–1,192; and the residues between the last helix and the ER retrieval motif at the C terminus, residues 1,474–1,510), this structure has disordered regions in the center of the TRXL2 domain (missing residues 461–505); and at the boundary between TRXL3 and TRXL4 (missing residues 862–886). The resolution of the *Ct*UGGT_Kif_ crystal structure is nevertheless sufficient to reveal that the molecule adopts a so far unobserved conformation, which we label “new-intermediate.” Figure 1D shows a superimposition of the “open” conformation (TRXL2 and TRXL3 domains in green) with the “new-intermediate” conformation (TRXL2 and TRXL3 domains in magenta). The *Ct*UGGT_kif_ “new-intermediate” conformation combines a TRXL1-TRXL3 distance as found in the “open” conformation and a TRXL2/TRXL3 relative orientation similar to the one found in the “closed-like” conformation. In what follows, we refer to the volume at the center of the UGGT molecule (between the βS1-βS2:GT24 portion and the TRXL2 domain) as the “central saddle;” the volume between the TRXL1 and TRXL3 domains are referred to as the “cleft” (Figure 2A).

To establish a framework for the discussion of UGGT inter-domain motions, we define here three collective conformational coordinates (CCs) (Figures 2A–2D). “CC1,” or “clamping,” measures the distance between the centers of mass of the TRXL1 and TRXL3 domains and the openness of the cleft between them (Figure 2B). “CC2,” or “bending,” measures the angle between the centers of mass of the TRXL1, TRXL2, and TRXL3 domains, and the proximity of the TRXL2 and GT24 domains across the central saddle (Figure 2C). Lastly, “CC3,” or “twisting,” measures the dihedral angle between the Cα atoms of residues *Ct*UGGT Y518, F466, T863, and I735 (the first two residues in the TRXL2 and the last two in the TRXL3 domain). Thus, the extent of UGGT twisting informs on the relative orientation of the TRXL2 and TRXL3 domains (Figure 2D).

Table 1 reports the values of the CCs for the conformations observed in *Ct*UGGT X-ray structures. The TRXL1:TRXL3 domain clamp is open in the “new-intermediate” *Ct*UGGT_Kif_ structure (CC1 = 43.2 Å). In the same structure, the TRXL2:TRXL3 domain pair twist adopts a middle-of-the-range value (CC3 = 3.2°). The pair of CC1/clamping and CC3/twisting values for the “new-intermediate” *Ct*UGGT_Kif_ structure—compared with the values of CC1 and CC3 in previously determined *Ct*UGGT structures—suggest that UGGT clamping and twisting motions may be to an extent independent of one another.

**Table 1:** Values of the conformational coordinates for the conformations observed in *Ct*UGGT X-ray structures and in extreme MD conformations

*Ct*UGGT PDB ID	Conformation	CC1 (Å)	CC2 (°)	CC3 (°)
5MZO	open	41.5	123.9	−13.1
5MU1	intermediate	37.5	133.0	−13.4
6TRF	intermediate	43.2	102.8	3.7
5N2J	closed	36.9	115.5	22.2
*Ct*UGGT^D611C/G1050C^, 5NV4	closed	37.1	116.4	−0.7
*Ct*UGGT^S180C/T742C^, 6TRT	closed	35.1	119.1	48.8
MD “W”	closed	40.3	88.7	9.5
MD “X”	open	47.0	125.0	−9.7
MD “Y”	open	38.6	148.8	−2.6
MD “Z”	open	37.7	143.8	15.9

### UGGT's motions can be described in simple terms as two rigid groups of domains moving with respect to each other

**Figure 3:**
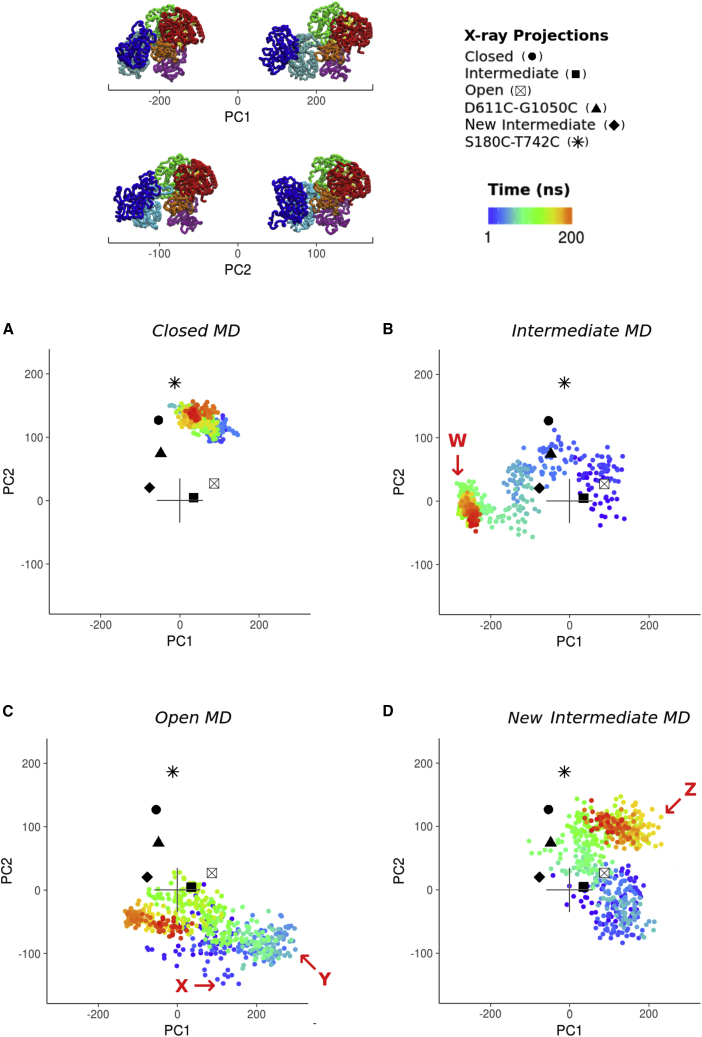
*Ct*UGGT projected MD trajectories Projections of individual MD trajectories and their respective X-ray starting structures onto the full conformational landscape as described by the first and second PCs, colored as a function of time. Domains colored as in Figures 1A and 1B. (A) MD trajectory projection starting from the “closed structure.” (B) MD trajectory projection starting from the “intermediate structure.” (C) MD trajectory projection starting from the “open structure.” (D) MD trajectory projection starting from the “new-intermediate structure.” In red, we list a few *Ct*UGGT structures representative of extreme values of the conformational coordinates, as identified within the MD conformational landscape (see Figure 4A). Figures made in RStudio (Team, 2016).

Next, we asked whether the conformational landscape spanned by *Ct*UGGT full-length crystal structures can be extended by *in silico* MD. We performed 250-ns long MD simulations starting from four of the *Ct*UGGT crystal structures (Figures 1E, 1F, 3, and S3; Videos S2 and S3). Compared with the set of crystal structures, UGGT MD trajectories do indeed span a wider conformational landscape. Principal components (PCs, also called essential modes [Capece et al., 2008]) were computed from the four individual MD trajectories and from the fusion of all four MDs into a single trajectory. Overall, UGGT's motions can be described in simple terms as two rigid groups of domains moving with respect to one another. One group is formed by domains TRXL2-TRXL3 and the other is formed by domains TRXL1-TRXL4-βS1-βS2-GT24—the latter group is enclosed in a gray circle in Figure 2A. The interface between domains TRXL3 and TRXL4 acts as a hinge region between the two domain groups.

Video S2: *Ct*UGGT MD PC1, related to Figure 2 The first PC of the joint MD simulation describes the transition between “open” and “closed” states and follows domain TRXL2 bending toward domain βS2 across the central saddle, with TRXL3 and TRXL1 clamping together across the cleft at the same time. Domains colored as in Figure 2.

Video S3: *Ct*UGGT MD PC2, related to Figure 2 The second PC of the joint MD simulation describes a movement in which the TRXL2 domain rotates with respect to TRXL3, and the βS2, TRXL1, and TRXL4 domains also undergo motion. Domains colored as in Figure 2.

The first two principal components of the joint MD simulation (PC1 and PC2) are illustrated in Figures 1E, 1F, and 3. PC1 and PC2 suffice to parameterize most of the observed motion. PC1 describes the transition between “closed” and “open” states and follows domain TRXL2 bending toward domain βS2 across the central saddle, with TRXL3 and TRXL1 clamping together across the cleft at the same time (Figures 1E and 3; Video S2). Figures 3B and 3C show that the MD simulations starting from the “intermediate” and “open” crystal structures both move significantly along PC1 and visit both “open” and “closed” states. The MD simulation starting from the “intermediate” structure drifts to the “closed” state and beyond, reaching very open conformations (Figure 3B). The MD simulation starting from the “open” structure shows a back-and-forth movement along PC1 (Figure 3C).

PC2 describes a movement in which the TRXL2 domain rotates with respect to TRXL3 (Figures 1F and 3; Video S3), with the βS2, TRXL1, and TRXL4 domains also undergoing motion. The motion encoded by PC2 is well represented in the MD starting from the “new-intermediate” *Ct*UGGT_Kif_ structure, whose projection in Figure 3D also shows a considerable degree of back-and-forth movement.

### Does the UGGT catalytic domain detach from the rest of the molecule?

The *Ct*UGGT βS1-βS2:GT24 portion of the molecule behaving as one relatively rigid structure throughout the MD simulations is hardly a surprise. The βS1-βS2:GT24 interface buries a 1,400-Å^2^ surface, with a calculated −7.1 kcal/mol solvation free energy gain (Krissinel, 2015). The βS1-βS2:GT24 interface is supported by 16 hydrogen bonds, five salt bridges, and 11 hydrophobic interactions, involving 86 residues overall (Figure S1A). The PISA server Complex Formation Significance Score is 1.0 (Krissinel, 2015), suggesting that the contacts in the *Ct*UGGT βS1-βS2:GT24 interface are sufficient to support the physiological nature of the observed N_term_:C_term_ inter-domain structure. The solvation free energy gain computed by the same server has a p value of 0.326. p < 0.5 indicates interfaces with higher than average hydrophobicity, implying that the interface is likely interaction specific (Krissinel, 2015).

The tight association we observe between the GT24 and βS1-βS2 domains is at odds with a hypothesis formulated on the basis of negative-stain electron microscopy (EM) and atomic force microscopy (AFM) of *Thermomyces dupontii* UGGT (*Td*UGGT) (Satoh et al., 2017; Satoh and Kato, 2018). That study proposed that the UGGT GT24 domain assumes a number of different relative orientations with respect to the rest of the molecule, enabled by the flexible linker between the βS2 and GT24 domains. No full-length crystal structure is available for *Td*UGGT. Based on sequence conservation, the GT24:βS1-βS2 interface of *Td*UGGT is also likely to be stable: of the 48 residues in the UGGT βS1-βS2:GT24 interface, 44 are conserved between *Td*UGGT and *Ct*UGGT, and none of the four residue differences would likely abrogate contributions to the GT24:βS1-βS2 interface (see Figure S1A). Hydrogen-deuterium exchange mass spectrometry (HDX-MS) data measured in solution for *Drosophila melanogaster* UGGT (*Dm*UGGT) (Calles-Garcia et al., 2017) also support solvent inaccessibility of the residues buried in the *Dm*UGGT βS1-βS2:GT24 interface (Figure S1B). These data taken together prompt the hypothesis that the GT24 and βS1-βS2 domains constitute a rigid group in *Td*UGGT also (and, by extension, in UGGTs across all eukaryotes), just as observed in our full-length *Ct*UGGT structures and MD simulations.

In the absence of a full-length *Td*UGGT crystal structure, the only information about the relative orientation of *Td*UGGT GT24 and βS1-βS2 domains comes from a 25-Å negative-stain EM reconstruction of *Td*UGGT in complex with an anti-*Td*UGGT antibody fragment (Fab) (Satoh et al., 2017; Satoh and Kato, 2018). To check whether the *Td*UGGT negative-stain EM reconstruction is compatible with a model in which GT24 and βS1-βS2 domains also form a rigid group, we generated a full-length *Td*UGGT homology model. We also selected a representative Fab structure from the Protein Data Bank (PDB). We then fitted the *Td*UGGT and Fab models both to the 25-Å negative-stained EM map for the complex of *Td*UGGT with its Fab and (separately) to its enantiomeric mirror image (Robert and Gouet, 2014). The *Td*UGGT:Fab models fitted to the original and inverted hands have been deposited in the PDB-DEV database (accession code PDBDEV_00000054). The correlation coefficients between the 25-Å negative-stained EM map and the *Td*UGGT:Fab models are around 90% for the fits to the original (Figures S2A–S2C) and the inverted hand map (Figures S2D–S2F) for both *Td*UGGT and Fab models. In the fitted models, the Fab contacts the 440–460 portion of *Td*UGGT domain TRXL2, in agreement with the published Fab epitope (residues *Td*UGGT 29–468) (Satoh et al., 2017; Satoh and Kato, 2018). In conclusion, the 25-Å negative-stained EM map of the complex of *Td*UGGT with its Fab can be fitted by a full-length *Td*UGGT model without invoking any detachment of the catalytic domain from the βS1-βS2 region, contrary to what is stated in Satoh et al. (2017) and Satoh and Kato (2018).

### UGGT inter-domain conformational mobility spans a wide range of conformations

**Figure 4:**
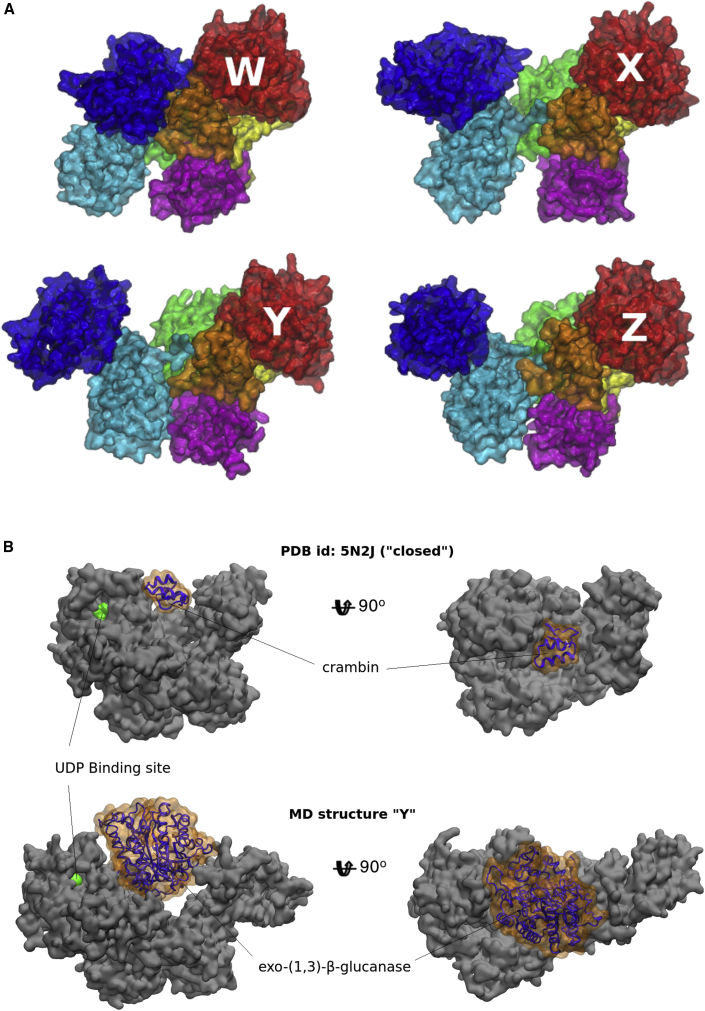
MD snapshots with extreme PC values The UGGT “one-size-fits-all adjustable spanner” model. (A) A few *Ct*UGGT structures representative of extreme values of the conformational coordinates (CCs), as identified within the MD conformational landscape. W, “clamped, bent, and twisted shut” (small values of CC1, CC2, and CC3). X, “clamped open” and “twisted open” (large CC1 and CC3 values); Y and Z, “clamped shut” (smaller values of CC1) but “bent open” (large CC2 values). Domains colored as in Figures 1A and 1B. (B) Two *Ct*UGGT conformations in complex with experimentally validated substrates of different sizes. The bright-green region shows the active site. Upper panel: crambin in complex with *Ct*UGGT “closed” crystal structure, conformation, MD-derived structure *Ct*UGGT “W” of (A). Lower panel: exo-(1,3)-β-glucanase in complex with MD-derived structure *Ct*UGGT “Y” of (A). Figures made in VMD (Cross et al., 2009).

As shown in Figure 3, MD simulations take *Ct*UGGT beyond the space sampled by the X-ray structures. In particular, the MD simulations starting from the “open,” “intermediate,” and “new-intermediate” *Ct*UGGT_Kif_ structures reach conformations with extreme PC values (Figure 4A and Table 1). Most notably, the structure labeled “W” in Figure 4 represents an extreme version of a closed state. It reveals that the seven UGGT domains can converge to a conformation of very compact overall shape. At the opposite end of the UGGT conformational landscape, structures labeled “X,” “Y,” and “Z” resemble extreme open-like states. Structure “X” in particular presents a notable opening of the TRXL1-TRXL3 cleft along the clamping motion described by CC1, while also showing a considerable degree of twisting along CC3. In contrast to “X,” structures “Y” and “Z” both exhibit a clamped cleft, but at extreme CC2 values. These MD conformations suggest that UGGT is able to push the bending motion even further than observed in the “open” structure while at the same time retaining a clamped cleft.

Measurements of the central saddle surface area in the observed UGGT MD conformations, from the most compact structure, “W,” to the most open structure, “Y,” span the range 8,600–11,300 Å^2^, with average values around 9,200–9,700 Å^2^ for most conformations (Video S4). Substrate glycoproteins with a “radius of gyration” (ROG) ⪟15 Å and around 150–200 residues or less would snugly fit in the central saddle of compact or middle-of-the-range UGGT conformers (Table S1 and Figure 4B, upper panels). In contrast, for binding of larger substrates (15 Å ⪟ ROG ⪟ 23 Å, and 200–500 residues), an opening of the central saddle would be needed (Table S1 and Figure 4B, lower panels).

Video S4: *Ct*UGGT MD snapshots, related to Figure 4 Snapshots from the MD simulations of *Ct*UGGT (gray), from a closed to an open conformation, with the residues contributing to the saddle surface colored according to the domain to which they belong (see Figure 2).

### *In vitro* monomeric UGGT activity implies the existence of the Parodi limit

Importantly, irrespective of the misfolded glycoprotein substrate, the finite size of UGGT puts an upper limit to the maximum distance between a site of misfold and an N-linked glycan that monomeric UGGT can reglucosylate on the same glycoprotein substrate *in vitro*. We propose the name “Parodi limit,” in honor of Armando J. Parodi (Parodi, 2007), for the maximum distance between a site of misfolding and an N-linked glycan that can be reglucosylated by monomeric UGGT *in vitro*, in response to recognition of misfold at that site. On the basis of our *Ct*UGGT MD simulations at 300 K and on the conformational mobility of Man_9_GlcNAc_2_ N-linked glycans (Mackeen et al., 2009), we estimate the Parodi limit to be in the region of 70–80 Å.

**Figure 5:**
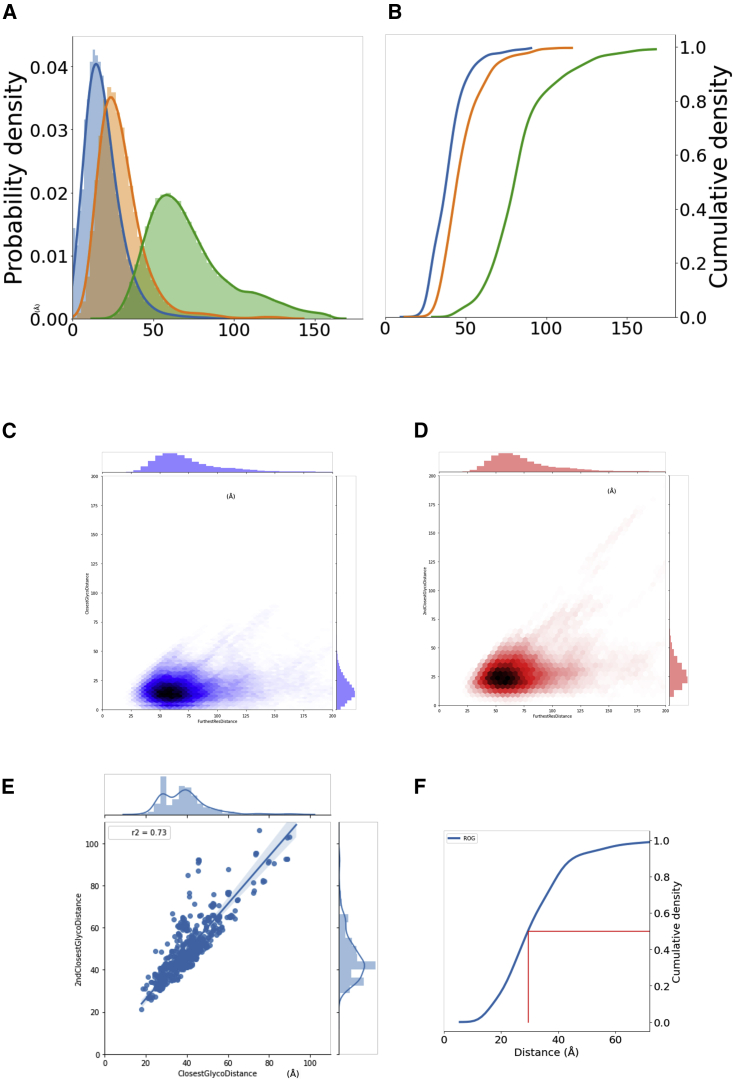
Frequency distributions of distances between glycoprotein residues and their nearest and second-nearest N-linked glycan Frequency distributions calculated over a sample of 1,244 glycoprotein crystal structures in the PDB. (A) Blue and orange: frequency distribution of the distance from every amino acid to its closest and second-closest N-linked glycan, respectively; green: frequency distribution of all unique aminoacid-aminoacid distances in the same structures. (B) Cumulative distributions of the distances in (A) (same color codes). (C) Frequency distribution of the distance from every amino acid to its closest N-linked glycan versus frequency distribution of all unique aminoacid-aminoacid distances. (D) Frequency distribution of the distance from every amino acid to its second-closest N-linked glycan versus frequency distribution of all unique aminoacid-aminoacid distances. (E) Correlation between the distances to the closest and second-closest glycan. (F) Cumulative distribution of the radius of gyration (ROG) of the glycoproteins. Red lines indicate ROG higher than or equal to that in 50% of the structures.

The relevance of this limit to UGGT *in vivo* activity remains to be elucidated. If UGGT acts as a monomer *in vivo* and does not rely on partner proteins in the ER to recognize its clients, the Parodi limit would impose evolutionary pressure on glycoproteins to evolve glycosylation sites within the same distance from their folding “Achilles' heels.” To probe this hypothesis, we have analyzed a sample of 1,244 glycoproteins structures in the PDB. The frequency distributions of the distance between every amino acid and its closest (and second-closest) N-linked glycan in these proteins are illustrated in Figure 5, together with the frequency distribution of aminoacid-aminoacid distances in the same structures (as a control). It is apparent that 99.61% of residues in these glycoproteins are closer to an N-glycosylation site than the Parodi limit and that this cannot be explained simply in terms of average glycoprotein size (Figures 5A–5D).

### UGGT activity depends on its inter-domain conformational mobility

In UGGT-mediated reglucosylation assays of urea-misfolded bovine thyroglobulin, both *Ct*UGGT^N796C/G1118C^ and *Ct*UGGT^D611C/G1050C^ mutants had lower activity than wild-type *Ct*UGGT, while *Ct*UGGT^N796C/G1118C^ had a higher catalytic activity and a lower melting temperature than *Ct*UGGT^D611C/G1050C^ (Roversi et al., 2017). Due to the extra disulfide bridge, *Ct*UGGT^N796C/G1118C^ cannot attain the “open” state, while *Ct*UGGT^D611C/G1050C^ can attain neither the “open” nor the “intermediate” conformation. As evidenced in Figure S3A, the MD trajectory starting from the *Ct*UGGT^D611C/G1050C^ structure shows significantly restricted mobility along the first PC, confirming that the extra disulfide bridge in *Ct*UGGT^D611C/G1050C^ tethers the TRXL2 and βS2 domains in a closed conformation. Along the second PC, *Ct*UGGT^D611C/G1050C^ moves further than the other double Cys mutants. The *Ct*UGGT^N796C/G1118C^ mutant, on the other hand, still retains most of its mobility, being able to explore a similar conformational space as those observed for wild-type *Ct*UGGT (Figure S3B). Taken together, these results suggest that the “bending” motion is important for reglucosylation of this particular substrate.

**Figure 6:**
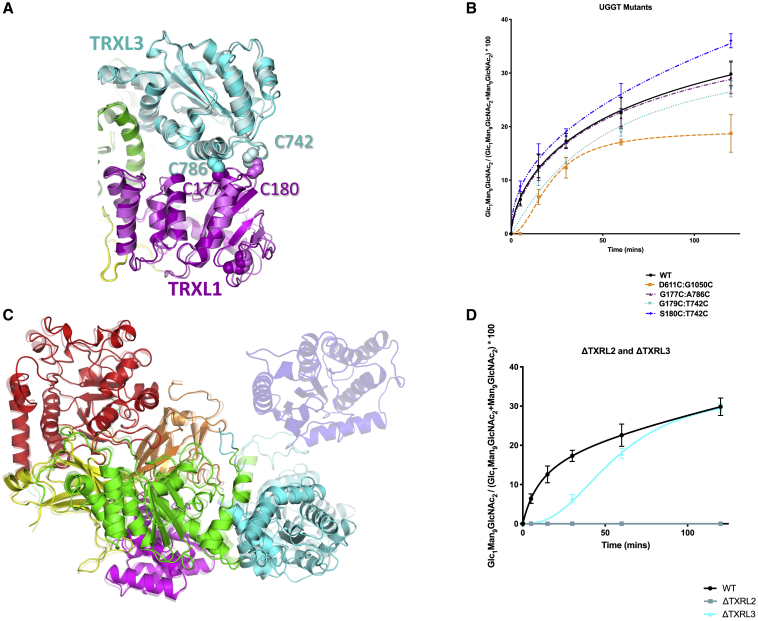
*Ct*UGGT double Cys and truncation mutants (A) The TRXL1 (magenta) and TRXL3 (cyan) domains in the crystal structures of *Ct*UGGT^G177C/A786C^ (PDB: 6TS8, dark colors) and *Ct*UGGT^S180C/T742C^ (PDB: 6TRT, lighter colors). The disulfide bonds are in sphere representation. (B) Reglucosylating activity of *Ct*UGGT double Cys mutants and wild-type (WT) *Ct*UGGT against urea-misfolded bovine thyroglobulin (mean values and standard deviation over three independent replicas). (C) Crystal structure of *Ct*UGGT-ΔTRXL2 (PDB: 6TS2, copy “A,” solid colors) overlaid onto wild-type *Ct*UGGT (“open” conformation, PDB: 5MZO, semi-transparent). Domains colored as in Figures 1A and 1B. (D) Activity of *Ct*UGGT-ΔTRXL2 and *Ct*UGGT-ΔTRXL3 against urea-misfolded bovine thyroglobulin, compared with wild-type (WT) *Ct*UGGT (mean values and standard deviation over three independent replicas). (A) and (C) made in PyMOL (Rigsby and Parker, 2016).

To probe the functional role of the “clamping” motion uncovered in the present analysis, we engineered four double-cysteine *Ct*UGGT mutants, *Ct*UGGT^G178C/A786C^, *Ct*UGGT^G177C/A786C^, *Ct*UGGT^G179C/T742C^, and *Ct*UGGT^S180C/T742C^, all designed to form disulfide bridges across the TRXL1 and TRXL3 domains, clamping shut the cleft between them. The *Ct*UGGT^G178C/A786C^ failed to express and was not studied any further. The presence of the engineered disulfide bridges in the remaining three mutants was confirmed by mass spectrometry (Figure S4). The crystal structures of *Ct*UGGT^G177C/A786C^ and *Ct*UGGT^S180C/T742C^ were determined to about 4.6-Å resolution. Both crystal structures show the TRXL3 domain tethered to the TRXL1 domain by the extra disulfide bridge (Figure 6A). We tested the *in vitro* activity of the three “clamped-shut” double Cys mutants (in addition to the activity of the wild type and the already published *Ct*UGGT^D611C/G1050C^) on urea-misfolded bovine thyroglobulin. Despite their structural similarity, the *Ct*UGGT^S180C/T742C^ and *Ct*UGGT^G177C/A786C^ mutants differ significantly in their ability to reglucosylate urea-misfolded bovine thyroglobulin: the former is more active than wild-type *Ct*UGGT while the latter has activity similar to that of the wild type (Figure 6B).

### *Ct*UGGT-mediated reglucosylation of urea-misfolded bovine thyroglobulin requires the TRXL2 domain

To assay the contributions of individual UGGT TRXL domains to UGGT reglucosylating activity, we cloned three mutants of *Ct*UGGT, each lacking one of the TRXL1-3 domains: *Ct*UGGT-ΔTRXL1, lacking residues 42–224; *Ct*UGGT-ΔTRXL2, lacking residues 417–650; and *Ct*UGGT-ΔTRXL3, lacking residues 666–870. *Ct*UGGT-ΔTRXL1 did not express and was not studied further. *Ct*UGGT-ΔTRXL2 and *Ct*UGGT-ΔTRXL3 expressed and were purified. *Ct*UGGT-ΔTRXL2 also yielded crystals, enabling crystal structure determination by X-ray diffraction to 5.7-Å resolution. At this resolution, the *Ct*UGGT-ΔTRXL2 crystal structure most closely resembles the “closed” structure (root-mean-square deviation_Cα_ [RMSD_Cα_] 1.32 Å with PDB: 5NV4, over 975 residues) apart from a minor rearrangement of the TRXL3 domain, which moves away from the rest of the truncated molecule (Figure 6C). *Ct*UGGT-ΔTRXL2 and *Ct*UGGT-ΔTRXL3 reglucosylation activity assays against urea-misfolded bovine thyroglobulin detect impaired reglucosylation activity upon deletion of TRXL3 and complete loss of activity upon deletion of TRXL2 (Figure 6D).

## Discussion

Since the discovery of UGGT in 1989 (Parodi, 2007; Trombetta et al., 1989), UGGT activity studies have used a range of glycoprotein substrates (Trombetta et al., 1989; Taylor et al., 2004; Ritter and Helenius, 2000; Ritter et al., 2005), small-size glycosylated synthetic compounds (Totani et al., 2006, 2009), and chemically synthesized misfolded glycoproteins (Izumi et al., 2016a, 2016b; Kiuchi et al., 2018). In addition to glycoprotein monomers, UGGT also surveys the quaternary structure of glycoprotein oligomers and larger multi-glycoprotein complexes (Keith et al., 2005; Zhang et al., 2011; Gardner and Kearse, 1999). A comprehensive list of physiological UGGT substrate glycoproteins has not been compiled, and the molecular detail of UGGT:substrate interactions remains uncharacterized. Yet it is apparent that the enzyme is highly promiscuous. The UGGT_2_ isoform (only present in higher eukaryotes) is competent in reglucosylating glycopeptides (Takeda et al., 2016), suggesting duplication of the UGGT gene and evolution of two isoforms with separate pools of misfolded glycoprotein substrates. If this is the case, the “UGGT1-ome” and “UGGT2-ome” (defined as full lists of clients of UGGT_1_ and UGGT_2_, respectively [Tax et al., 2019]) would contain distinct (although possibly overlapping) sets of substrate glycoproteins. Still, each substrate glycoprotein potentially presents a unique relative orientation and distance between the site of misfold and the N-linked glycan receiving the glucose. How each UGGT isoform can reglucosylate misfolded glycoproteins of such a wide variety of different sizes and shapes therefore constitutes a major open question.

Our MD simulations of *Ct*UGGT confirm that—despite its tightly woven topology (Roversi et al., 2017)—the enzyme is indeed quite flexible. More importantly, our analysis of the observed UGGT MD conformational landscape establishes the framework necessary to discuss the enzyme's dynamics. The molecule's inter-domain conformational motions can be described in terms of three simple CCs: the relative movement between domains TRXL3 and TRXL1, resulting in the opening and closing of the cleft between them (“clamping,” along CC1); the movement restricted to TRXL2 moving closer or further away from the relatively rigid core composed of domains GT24-βS1-βS2-TRXL4 (“bending,” along CC2); and the rotation of TRXL2 with respect to TRXL3 (“twisting,” along CC3). The three motions are to some extent independent of each other. These observations open the way to the cloning, expression, and purification of Cys quadruple mutants such as *Ct*UGGT^G177C/A786C, D611C/G1050C^: these mutants would block the molecule in a clamped and bent closed conformation across the cleft and the central saddle, respectively. Equivalent mutants would likely aid structural studies of mammalian UGGTs, which so far have resisted structural determination (Parodi et al., 2014).

UGGTs recombinantly expressed or tissue-purified from eukaryotic cells have all so far revealed cleavage in the flexible linker between the folding sensor N-terminal portion and the catalytic GT24 domain (see a survey in Roversi et al., 2017). The one study speculating large relative movements between the two portions of the UGGT molecule thanks to this flexible linker (Satoh et al., 2017; Satoh and Kato, 2018) was based on bacterially expressed *T. dupontii* UGGT, *Td*UGGT. The GT24 and βS1-βS2 domains of *Td*UGGT in that study can indeed be pulled apart by AFM: this experiment likely induces mechanical denaturation, breaking the interface between these domains in a non-physiological manner. Here, we consulted all the available biochemical and structural evidence to test the hypothesis that the UGGT βS1-βS2 and GT24 domains also constitute a rigid unit in *Td*UGGT. We analyzed all crystal structures of full-length UGGTs and their mutants, their MD trajectories, HDX-MS data for *Dm*UGGT (Calles-Garcia et al., 2017), and the 25-Å negative-stained EM map for the complex between *Td*UGGT and an anti-*Td*UGGT Fab (Satoh et al., 2017; Satoh and Kato, 2018). We found no evidence suggesting separation of the βS1-βS2 and GT24 domains on either side of the cleaved flexible linker. Claims to the contrary in Satoh et al. (2017) and Satoh and Kato (2018) were likely due to difficulties in docking the N-terminal (PDB: 5Y7O) and C-terminal portions (PDB: 5H18) of *Td*UGGT separately into the negative-stain EM map, without any higher-resolution knowledge of a full-length UGGT structure. It is possible that the energy needed for disrupting the UGGT GT24:βS1-βS2 interface be supplied for example by ATP hydrolysis. Of course, if the cleavage at the UGGT flexible linker (observed in all eukaryotically expressed UGGT to date) is physiological, the two portions of the molecule would then fly apart upon disassociation.

Based on the data available, UGGT's promiscuity is not likely dependent on the flexible linker between the catalytic domain and the N-terminal misfold sensing region. Rather, it appears to be underpinned by the motions uncovered by the MD simulations. The question remains regarding UGGT's reported ability to survey not only folding of small- and medium-size glycoprotein monomers but also the quaternary structure of glycoprotein oligomers and larger multi-glycoprotein complexes (Keith et al., 2005; Zhang et al., 2011; Gardner and Kearse, 1999). The UGGT inter-domain movements (as uncovered by our MD simulations) extend beyond what was observed in the crystal structures. Indeed, *in silico* modeling suggests that the extended UGGT conformations sampled by MD could accommodate glycoprotein substrates of different sizes. Such extended conformations would enable UGGT reglucosylation across a wide range of distances between an N-glycosylation site and a site of misfold.

UGGT is active *in vitro* as a monomer (as judged by its size-exclusion chromatography elution volume [Roversi et al., 2017]). Activity of monomeric UGGT *in vitro* implies the existence of an upper bound to the distance between a site of misfold on a UGGT substrate and the closest N-linked glycan the enzyme can reglucosylate on the same substrate *in vitro*. We introduce the term “Parodi limit” for this upper bound, in honor of Armando J. Parodi (Parodi, 2007). On the basis of the most “open” conformations observed in the MD simulations carried out on *Ct*UGGT at 300 K, we estimate the Parodi limit to be close to or less than 70–80 Å. The current lack of knowledge about the UGGT site(s) of misfold recognition, the considerable conformational mobility of the N-linked glycan, and the unknown dependence of the UGGT conformational landscape on the temperature make it difficult to put an estimated error on this value. Functional data from UGGT-mediated reglucosylation of a series of rigid, misfolded UGGT glycoprotein substrates (each bearing one recombinantly engineered N-linked glycosylation site at a specific distance from a single site of misfold common to all substrates in the series) would enable experimental estimation of the Parodi limit. Ideally, one such series of artificial N-linked glycosylation sites at varying distances from a single site of misfold would have to be engineered for a number of different substrate glycoprotein scaffolds in order to minimize the dependency of the Parodi limit estimation from a given substrate series and enable estimation of a standard error on that value.

*In vivo/in cellula*, it is of course possible that UGGT misfolded glycoprotein recognition can be mediated by UGGT dimers/multimers or aided by UGGT ER partner proteins. One such candidate is the ER HSP70 BiP ATPase, which is found in ER multiprotein complexes with UGGT_1_ (Kastritis et al., 2017; Meunier et al., 2002). The physiological relevance of the Parodi limit to UGGT's activity *in vivo* therefore remains to be investigated. Existence of this limit *in vivo* would in turn imply evolutionary pressure on N-glycosylation sites to develop at accessible distances from the portions of a glycoprotein that are most prone to folding difficulties (i.e., the folding glycoprotein's Achilles’ heels). We made a first preliminary attempt at checking the distance distributions of N-linked glycans from glycoprotein residues, examining them in the light of our current estimation of the Parodi limit. In the absence of detailed knowledge on UGGT-omes, a sample of 1,244 glycoprotein structures in the PDB were analyzed. The analysis assumes glycoprotein crystal structures to be representative of the sizes/shapes of UGGT misfolded clients: UGGT efficiently reglucosylates only misfolded rather than fully unfolded glycoproteins. The majority of glycoproteins in this sample have at least one N-linked glycan within the Parodi limit from every amino acid, irrespective of glycoprotein size. These observations support the hypothesis that the constraints imposed by UGGT structure exert evolutionary pressure on the distribution of N-linked glycosylation sites in UGGT clients.

When it comes to correlating UGGT inter-domain conformational mobility with its activity, among the *Ct*UGGT double-cysteine mutants tested so far the *Ct*UGGT^D611C/G1050C^ mutant described in Roversi et al. (2017) is the least active in reglucosylating urea-misfolded bovine thyroglobulin. This observation is compatible with MD trajectory of this mutant being the most severely limited one across our simulations. The extra disulfide bridge engineered in this mutant joins the βS2-TRXL2 domains, giving rise to the hypothesis that during the enzyme:substrate encounter, a portion of misfolded thyroglobulin may be accommodated in the UGGT central saddle between these domains. The *Ct*UGGT crystal and cryo-EM structures (Roversi et al., 2017), and our MD simulations, all highlight the TRXL2 domain as the most mobile in the molecule, supporting this hypothesis. The total loss of activity of the *Ct*UGGT-ΔTRXL2 construct in the reglucosylation of urea-misfolded bovine thyroglobulin also points to a critical role for the TRXL2 domain. In the light of these data, it is possible that TRXL2 motions adjust the size or vary the surface area of UGGT's central saddle, making the bending motion a crucial one for activity against this substrate.

As to the UGGT clamping motion, we tested two a priori rather similar double-cysteine mutants, *Ct*UGGT^S180C/T742C^ and *Ct*UGGT^G177C/A786C^, both designed to clamp the TRXL1-TRXL3 domains shut. Surprisingly, the two mutants differ in their ability to reglucosylate urea-misfolded bovine thyroglobulin, with the former mutant more active than (and the latter mutant having similar activity to) wild-type *Ct*UGGT. These observations point to the possibility that each misfolded glycoprotein substrate may depend to a different degree on a different subset of UGGT inter-domain conformational degrees of freedom. In the light of these data, dissection of the UGGT structure/activity relationship will profit from a number of reglucosylation assays using the same set of UGGT mutants on different glycoprotein substrates.

The *Ct*UGGT-ΔTRXL3 mutant appears to be competent in reglucosylating urea-misfolded bovine thyroglobulin. Full kinetic characterization would be needed to further test the observed lower activity of the *Ct*UGGT-ΔTRXL3 mutant in terms of slower rate and/or lower substrate affinity. In contrast, *Ct*UGGT-ΔTRXL2 is completely inactive against urea-misfolded bovine thyroglobulin. Substrate recruitment via TRXL2 movements would not require complete burial of the misfolded glycoprotein into the central saddle of the molecule: UGGT would minimally need to establish contact with the portion of substrate containing the misfold. This is plausible for relatively big substrates such as transferrin (77 kDa, radius of gyration = 29.7 Å) or urea-misfolded bovine thyroglobulin (670 kDa, a long, presumably snake-like chain of eleven 60-amino-acid compact domains, no structure available). For smaller substrates, such as glycopeptides or synthetic fluorescent probes, more closed UGGT conformations, bringing TRXL2 toward βS2 and the GT24 domain across the central saddle, may be needed.

Apart from TRXL2, other untested UGGT regions potentially harboring exposed hydrophobic patches are the *Ct*UGGT TRXL4 disordered region (*Ct*UGGT 243–285), the flexible linker around the endo-proteolysis site between the βS2 and GT24 domains (*Ct*UGGT 1,153–1,195), and the residues between the last helix and the ER retrieval motif at the C terminus (*Ct*UGGT 1,474–1,510) (Roversi et al., 2017). Experiments described in a recent report ascribe intrinsic refoldase activity to UGGT (Wang et al., 2020) and await being reproduced. Again, structural and functional data from a range of UGGT mutants and glycoprotein substrates will be required to further test these hypotheses and fully dissect the UGGT structure-function relationship.

The molecular forces supporting UGGT-mediated glycoprotein misfold recognition have been generally hypothesized to be hydrophobic interactions (Caramelo et al., 2003). Our observation that the face of the TRXL2 domain overlooking the central saddle bears distinct patches of hydrophobic residues conserved across UGGT_1_ sequences (Roversi et al., 2017) supports a model of hydrophobic-mediated misfold recognition. Dependency of misfold recognition on disordered portions of a misfold checkpoint enzyme was observed for the mouse ERAD mannosidase (EDEM), which also preferentially acts on misfolded glycoproteins and was proved to undergo constant ERAD degradation itself (Marin et al., 2012). The hypothesis that UGGT works by having evolved an intrinsically misfolded portion, with which the enzyme would interact with substrate glycoprotein misfolded regions, would in turn imply that UGGT may reglucosylate itself. If this is the case, UGGT would also be likely subjected to constant ERAD demannosylation and degradation. The fact that UGGT bears demannosylated glycans—a hallmark of ERAD (Daikoku et al., 2014, 2015)—is compatible with this hypothesis. If indeed the ERAD and ERQC checkpoint enzymes recognize misfolded glycoproteins via an intrinsically misfolded domain (“it takes one to know one” [Tax et al., 2019]), the associated biochemical costs of this strategy may be the price eukaryotic cells pay in order to afford UGGT/EDEMs as broad-specificity glycoprotein misfolding checkpoints. *In vitro* and *in cellula* experiments to test these ideas are in progress.

## STAR★Methods

### Key resources table

**Table:** 

REAGENT or RESOURCE	SOURCE	IDENTIFIER
**Bacterial and virus strains**		

*E*.*coli* DH5-α	New England Bioscience	Cat# C2987I
*E*.*coli* BL21	New England Bioscience	Cat# C2530H
XL10-Gold Ultracompetent cells	Agilent	Cat# 200317

**Chemicals**, **peptides**, **and recombinant proteins**		

Q5® Hot Start High-Fidelity 2X Master Mix	New England Biolabs	Cat# M0494S
NEBuilder® HiFi DNA Assembly Master Mix	New England Biolabs	Cat# E2621S
Gibson Assembly Kit	New England Biolabs	Cat# E2611
Bovine Thyroglobulin	Sigma-Aldrich	Cat# T1001
KLD Enzyme Mix	New England Biolabs	Cat# M0554S
KLD Reaction Buffer	New England Biolabs	Cat# M0554S
QIAprep Spin Miniprep Kit (Qiagen)	QIAGEN	Cat# 27104
EndoFree Plasmid Kits	QIAGEN	Cat# 12362
QIAquick gel extraction kit	QIAGEN	Cat# 28706
Cutsmart^TM^ buffer	New England Biolabs	Cat# B7204S
AgeI	New England Biolabs	Cat# R0552S
KpnI	New England Biolabs	Cat# R0142S
PNGase F	New England Biolabs	Cat# P0704S
PNGase glycobuffer 2	New England Biolabs	Cat# B7002S
Denaturing buffer	New England Biolabs	Cat# B1704S
Anthranilic Acid	Sigma-Aldrich	Cat# A89855
OptiPRO™ SFM	ThermoFisher Scientific	Cat# 12309019
FreeStyle^TM^ 293 Expression Medium	ThermoFisher Scientific	Cat# 12338001
FreeStyle^TM^ MAX transfection reagent	ThermoFisher Scientific	Cat# 16447100
SOC Media	New England Biolabs	Cat# B9020S
Kifunensine	Cayman Chemical	Cat# 109944-15-2
Carbenicillin	Sigma-Aldrich	Cat# C1389
*Ct*UGGT	Ref. (Roversi et al., 2017)	N/A
*Ct*UGGT^S180C/T742C^ protein	This paper	N/A
*Ct*UGGT_Kif_ protein	This paper	N/A
*Ct*UGGT-ΔTRXL2 protein	This paper	N/A
*Ct*UGGT^G177C/A786C^ protein	This paper	N/A
Imidazole	Honeywell Fluka	Cat# 56750
HEPES	Sigma-Aldrich	Cat# H3375
MORPHEUS Crystallisation Screen	Molecular Dimensions	Cat# MD1–47
MORPHEUS2 Crystallisation Screen	Molecular Dimensions	Cat# MD1-92
JCSG+ Crystallisation Screen	Molecular Dimensions	Cat# MD1–40

**Critical commercial assays**		

Sequencing Grade Modified Trypsin	Promega	Cat# V5111

**Deposited data**		

*Ct*UGGT^G1050C/D611C^ mutant	(Roversi et al., 2017)	PDB ID: 5NV4
*Ct*UGGT ‘closed’	(Roversi et al., 2017)	PDB ID: 5N2J
*Ct*UGGT ‘open’	(Roversi et al., 2017)	PDB ID: 5MZO
*Ct*UGGT ‘intermediate’	(Roversi et al., 2017)	PDB ID: 5MU1
*Ct*UGGT^S180C/T742C^ mutant	This paper	PDB ID: 6TRT
*Ct*UGGT_Kif_ mutant	This paper	PDB ID: 6TRF
*Ct*UGGT-ΔTRXL2 mutant	This paper	PDB ID: 6TS2
*Ct*UGGT^G177C/A786C^ mutant	This paper	PDB ID: 6TS8
*Td*UGGT:Fab model	This paper	PDBDEV 00000054

**Experimental models**: **cell lines**		

HEK FreeStyle^TM^ 293F cells	ThermoFisher Scientific	Cat# R79007

**Oligonucleotides**		

Primers for Gibson Assembly	See Table S4	N/A
Primers for mutagenesis to obtain CtUGGT_ΔTRXL1	See Table S4	N/A
Primers for mutagenesis to obtain CtUGGT_ΔTRXL2	See Table S4	N/A
Primers for mutagenesis to obtain CtUGGT_ΔTRXL3	See Table S4	N/A
Primers for mutagenesis to obtain CtUGGT^G177C^ mutation	See Table S4	N/A
Primers for mutagenesis to obtain CtUGGT^V178C^ mutation	See Table S4	N/A
Primers for mutagenesis to obtain CtUGGT^S180C^ mutation	See Table S4	N/A
Primers for mutagenesis to obtain CtUGGT^T742C^ mutation	See Table S4	N/A
Primers for mutagenesis to obtain CtUGGT^A786C^ mutation	See Table S4	N/A

**Recombinant DNA**		

*Ct*UGGT-pHLsec plasmid	Ref. (Roversi et al., 2017)	N/A
*Ct*UGGT^S180C/T742C^ -pHLsec plasmid	This paper	N/A
*Ct*UGGT_Kif_ -pHLsec plasmid	This paper	N/A
*Ct*UGGT-ΔTRXL2 -pHLsec plasmid	This paper	N/A
*Ct*UGGT^G177C/A786C^ -pHLsec plasmid	This paper	N/A

**Software and algorithms**		

Empower	Waters Inc.	Version 3.0
AMBER suite	https://ambermd.org/	Version18
Clustal omega	https://www.ebi.ac.uk/Tools/msa/clustalo/	Version 1.2.4
Modeller	http://salilab.org/modeller/	Version 9.19
VMD	(Cross et al., 2009)	Version 1.9.3
RStudio Desktop	RStudio Team (2020). RStudio: Integrated Development for R. RStudio, PBC, Boston, MA URL http://www.rstudio.com/. (RStudio Team, 2016)	Version 1.2.5042
MassMatrix Suite 1.3.3	https://massmatrix.bio/	Version 2.4.2
MSconvert from the ProteoWizard toolbox	http://proteowizard.sourceforge.net/	Version 3.3.19172-57d620127
autoBUSTER	(Blanc et al., 2004)	Version 2.10.3
Phaser	(McCoy et al., 2007)	Version 2.8.3
autoPROC	(Vonrhein et al., 2011)	Version 1.0.5
Coot	(Emsley et al., 2010)	Version 0.9
Refmac5	(Nicholls et al., 2012)	Version 5.8.0258
Molrep	(Vagin and Teplyakov, 2010)	Version 11.7.02
Chimera	(Pettersen et al., 2004)	Version 1.14
GraphPad Prism	GraphPad Software, San Diego, California USA, www.graphpad.com	Version 8.0.0 for Windows

### Resource availability

#### Lead contact

Further information and requests for resources and reagents should be directed to and will be fulfilled by the Lead Contact, Pietro Roversi (pr159@leicester.ac.uk).

#### Materials availability

Plasmids generated in this study will be made available on request by the Lead Contact with a completed Materials Transfer Agreement (MTA).

#### Data and code availability

The X-ray crystallographic data and atomic models have been deposited at the Protein Data Bank. The accession number for the *Ct*UGGT_Kif_ crystal structure reported in this paper is PDB: 6TRF. The accession number for the *Ct*UGGT-ΔTRXL2 crystal structure reported in this paper is PDB: 6TS2. The accession number for the *Ct*UGGT^S180C/T742C^ crystal structure reported in this paper is PDB: 6TRT. The accession number for the *Ct*UGGT^G177C/A786C^ crystal structure reported in this paper is PDB: 6TS8. The models for the *Td*UGGT:Fab complex (fitted in the original hand and the inverted hand of the negative stain EM reconstruction) have been deposited in PDB-DEV. The accession number for the *Td*UGGT:Fab complex reported in this paper is PDBDEV: 00000054.

A list of pieces of software used in this study can be found in the Key Resources Table.

### Experimental model and subject details

#### Bacteria

Commercial *E*. *coli* DH-5α chemically competent cells and BL21 cells were purchased from New England Biolabs (NEB) and handled as per manufacturer’s instructions (Cat# C2987I and Cat# C2530H). XL10-Gold Ultracompetent cells were purchased from Agilent and handled as per manufacturer’s instructions (Cat# 200317).

#### Cell lines

Human epithelial kidney FreeStyle 293F cells (ThermoFisher Scientific) were cultured in FreeStyle 293 Media (ThermoFisher Scientific) in Erlenmeyer flasks with 0.2 μm vent caps (Corning) shaking at 135 revolutions per min (rpm) in a 37 °C incubator kept at 8% CO_2_.

### Methods details

#### Cloning

All DNA primers were purchased from Sigma. Details of the cloning of full-length *Ct*UGGT are described in (Roversi et al., 2017). All *Ct*UGGT mutants were generated starting from the gene of *Ct*UGGT inserted in Litmus28i (an optimal vector for mutagenesis experiments), using Q5® Hot Start High-Fidelity 2X Master Mix (New England Biolabs - NEB) following manufacturer instructions; briefly: 12.5 μL of Q5® Hot Start High-Fidelity 2X Master Mix (New England Biolabs) were added to 1.25 μL of each forward and reverse primer at 10 μM, 1 μL of *Ct*UGGT:Litmus28i DNA at 1 ng/μL and 9 μL of nuclease-free water, obtaining a 25 μL final volume. To generate the double cysteine mutants, the DNA obtained from the first mutation was used as starting material for the second mutation insertion. PCR amplification was then performed with a personalised protocol for each mutant, as described in detail further on in this section. Kinase, Ligase & DpnI (KLD) treatment: 1 μL of PCR product was mixed with 5 μL of 2X KLD Reaction buffer, 1 μL of 10X KLD Enzyme Mix (both from NEB) and 3 μL of nuclease-free water. The mixture was incubated at room temperature for 5 minutes. The KLD reaction mixture was used to transform *E*. *coli* DH-5α chemically competent cells (NEB) using the following protocol: 5 μL of KLD reaction mix were added to a tube of thawed DH-5α competent *E*. *coli* cells on ice, and mixed gently for a few seconds; after transformation, the bacteria were incubated on ice for 30 minutes, heat shocked at 42 °C for 30 seconds and incubated on ice again for 5 minutes. 950 μL of SOC media (New England Biolabs) were added to a final volume of 1 mL and the mixture was incubated for 1 hour at 37 °C with gentle shaking at 200/300 rpm. 100 μL of the bacteria were spread onto a pre-warmed (37 °C) LB agar culture plate containing carbenicillin (Sigma-Aldrich, 0.1 mg/mL). The plate was incubated at 37 °C overnight. Colony-PCR was performed on DNA from various colonies by using T7_F (5’-TAATACGACTCACTATAGGG-3’) and T7_R (5’-GCTAGTTATTGCTCAGCGG-3’) primers and the DNA obtained was loaded on a 1% w/v agarose gel and run for 50 minutes at 150 V. Analysis of this gel allowed identification of colonies with amplified DNA of the appropriate size; cells from colonies containing an amplified product of the desired size were used to inoculate 5 mL LB supplemented with 0.1 mg/mL carbenicillin. Following overnight incubation at 37°C, plasmid mini-preps were performed using the QIAprep Spin Miniprep Kit (Qiagen) according to the manufacturer's instructions. Glycerol stocks were obtained by mixing 16% glycerol with 84% bacteria, and freezing and storing at -80 °C. The DNA obtained was sequenced with the appropriate primers. The glycerol stock was used to inoculate 5 mL LB supplemented with 0.1 mg/mL carbenicillin and incubated over night at 37 °C. This culture was then used to inoculate 200 mL LB supplemented with 0.1 mg/mL carbenicillin and the bacteria were incubated at 37 °C and 110 rpm. Upon reaching an OD_600nm_ of 2.0, the cells were spun down at 3320xg for 18 minutes. The pellets were resuspended, and the plasmid maxi-preps were performed to purify the DNA using EndoFree Plasmid Kits (Qiagen), following the recommended protocol. The mutants in Litmus28i were subsequently cloned into the pHLsec expression vector (Aricescu et al., 2006) to contain a hexa-His Tag at the C-terminus. DNA for pHLsec was linearised using AgeI and KpnI restriction enzymes (NEB) at 37 °C for 16 hours in Cutsmart^TM^ buffer (NEB). The restriction digest was then run on a 0.8% w/v agarose gel at 150 V for 1 hour. The linearised vector was cut from the gel and purified with a QIAquick gel extraction kit (Qiagen). PCR was then performed on each *Ct*UGGT mutant in Litmus 28i as follow: 1 μL of DNA (1 ng/μL) added at 25 μL of Q5® Hot Start High-Fidelity 2X Master Mix (NEB), 2.5 μL of each forward (pHLsec_*Ct*UGGT_F: 5'-GGTTGCGTAGCTGAAACCGGTCAAGTCGCAGCCTCTCCA-3') and reverse (pHLsec_*Ct*UGGT_R: 5'-GATGGTGGTGCTTGGTACCCTCCCGAACCGTCTTGAC-3') primers and 19 μL of nuclease-free water. PCR protocol: step 1: 98 °C for 30 seconds; step 2: 98 °C for 10 seconds; step 3: 62 °C for 30 seconds; step 4: 70 °C for 150 seconds; step 2-4 were repeated 35 times; step 5: 72 °C for 2 minutes. The PCR products were run on a 0.8% w/v agarose gel at 150 V for 1 hour and the amplified insert was cut from the gel and purified with the same QIAquick gel extraction kit. A Gibson Assembly was then performed using the gel-purified PCR-amplified *Ct*UGGT mutant insert mixed with gel-purified linearised pHLsec at a ratio of 3:1 with NEBuilder® HiFi DNA Assembly Master Mix (NEB) using manufacturer suggested protocol, for 1 hour at 50 °C. 2 μl of this ligation product was added to 50 μl XL10-Gold Ultracompetent cells (Agilent), following the transformation guideline protocol. The cells were then plated on 0.1 mg/mL carbenicillin agar plates and incubated over night at 37 °C. Colony-PCR was performed on DNA from various colonies (using pHLsec_F and pHLsec_R primers) and the DNA obtained was run on a 1% w/v agarose gel for 50 minutes at 150 V. Analysis of this gel allowed identification of colonies with amplified DNA of the appropriate size; mini-prep, glycerol stock, DNA sequencing and maxi-prep were performed to obtain *Ct*UGGT mutant pHLsec plasmid DNA.

***Cloning of CtUGGT***^***A786C***^. Mutation of the *Ct*UGGT into *Ct*UGGT^A786C^ was carried out starting from the gene of *Ct*UGGT inserted in Litmus28i as described before, using forward (A786C_F: 5'-CGCTTACGACtgtTCTCTAGCCAAC-3') and reverse (A786C_R: 5'-ACATCTGGTTCGAACTCG-3') primers. PCR amplification: step 1: 98 °C for 30 s; step 2: 98 °C for 10 s; step 3: 60 °C for 20 s; step 4: 72 °C for 135 s. Steps 2-4 were repeated 25 times; step 5: 72 °C for 2 minutes. KLD treatment and *E*.*coli* transformation was performed as described before, and later mini and maxi-prep, as detailed above. *Ct*UGGT^A786C^:Litmus28i plasmid DNA, 3 mL at 400 ng/μL were obtained.

**Cloning of *CtUGGT***^***G177C/A786C***^. To obtain the double mutant *Ct*UGGT^G177C/A786C^, the second mutation G177C was introduced starting from the gene of *Ct*UGGT^A786C^ in Litmus28i as described above, using forward (G177C_F: 5'-TCGGAAGTTTtgcGTTGGTTCCC-3') and reverse (G177C_R: 5'-TCAAATGGCAGTGTCCGC-3') primers. PCR protocol: step 1: 98 °C for 30 seconds; step 2: 98 °C for 10 seconds; step 3: 66 °C for 30 seconds; step 4: 72 °C for 135 seconds; steps 2-4 were repeated 25 times. Step 5: 72 °C for 2 minutes. After KLD treatment (see above) *E*. *coli* DH-5α chemically competent cells were transformed with the DNA as described previously. Mini and maxi-prep, as detailed above, yielded 3 mL of *Ct*UGGT^G177C/A786C^:Litmus28i plasmid DNA at 700 ng/μL. The insert was then linearised, cloned into pHLsec vector (by Gibson Assembly) as described above, and after mini- and then maxi-prep, 3 mL of *Ct*UGGT^G177C/A786C^:pHLsec plasmid DNA at 300 ng/μL were obtained.

**Cloning of *CtUGGT***^***V178C/A786C***^. To obtain the double mutant *Ct*UGGT^V178C/A786C^, the second mutation V178C was introduced starting from the gene of *Ct*UGGT^A786C^ in Litmus28i as described above, using forward (V178C_F: 5'-GAAGTTTGGCtgtGGTTCCCGTG-3') and reverse (V178C_R: 5'-CGATCAAATGGCAGTGTC-3') primers. PCR protocol: step 1: 98 °C for 30 seconds; step 2: 98 °C for 10 seconds; step 3: 60 °C for 30 seconds; step 4: 72 °C for 135 seconds; steps 2-4 were repeated 25 times. Step 5: 72 °C for 2 minutes. After KLD treatment (see above) *E*. *coli* DH-5α chemically competent cells were transformed with the DNA as described previously. Mini and maxi-prep, as detailed above, yielded 3 mL of *Ct*UGGT^V178C/A786C^:Litmus28i plasmid DNA at 500 ng/μL. The insert was then linearised, cloned into pHLsec vector (by Gibson Assembly) as described above, and after mini- and then maxi-prep, 3 mL of *Ct*UGGT^V178C/A786C^:pHLsec plasmid DNA at 700 ng/μL were obtained.

***Cloning of CtUGGT***^***S180C/T742C***^. Mutation of the *Ct*UGGT into *Ct*UGGT^T742C^ was effected starting from the gene of *Ct*UGGT inserted in Litmus28i as described before, using forward (T742C_F: 5'-TCCCAAGGATtgcTCACGTTCCC-3') and reverse (T742C_R: 5'-TTGTGGACAATGTCCAAC-3') primers properly designed. PCR amplification: step 1: 98 °C for 30 s; step 2: 98 °C for 10 s; step 3: 59 °C for 20 s; step 4: 72 °C for 135 s. Steps 2-4 were repeated 25 times; step 5: 72 °C for 2 minutes. KLD treatment and *E*.*coli* transformation were performed as described before, and later mini and maxi-prep, as detailed above. *Ct*UGGT^T742C^:Litmus28i plasmid DNA, 3 mL at 500 ng/μL were obtained. To obtain the double mutant *Ct*UGGT^S180C/T742C^, the second mutation S180C was introduced starting from the gene of *Ct*UGGT^T742C^ in Litmus28i as described above, using forward (S180C_F: 5'-TGGCGTTGGTtgcCGTGATGTGA-3') and reverse (S180C_R: 5'-AACTTCCGATCAAATGGCAGTGTC-3') primers. PCR protocol: step 1: 98 °C for 30 seconds; step 2: 98 °C for 10 seconds; step 3: 68 °C for 30 seconds; step 4: 72 °C for 135 seconds; steps 2-4 were repeated 25 times. Step 5: 72 °C for 2 minutes. After KLD treatment (see above) *E*. *coli* DH-5α chemically competent cells were transformed with the DNA as described previously. Mini and maxi-prep, as detailed above, yielded 3 mL of *Ct*UGGT^S180C/T742C^:Litmus28i plasmid DNA at 500 ng/μL. The insert was then linearised, cloned into pHLsec vector (by Gibson Assembly) as described above, and after mini- and then maxi-prep, 3 mL of *Ct*UGGT^S180C/T742C^:pHLsec plasmid DNA at 700 ng/μL were obtained.

***Cloning of CtUGGT-ΔTRXL1***. The *Ct*UGGT-ΔTRXL1 construct lacks residues *Ct*UGGT 42-224. The deletion of the *Ct*UGGT TRXL1 domain was performed starting from the gene of *Ct*UGGT in Litmus28i as described before, using forward (Δ1_F: 5'-GAGTCTCTGTCCGTCAATGG-3') and reverse (Δ1_R: 5'-AGAGGGGAAAGCGGCTTT-3') primers properly designed. PCR protocol: step 1: 98 °C for 30 seconds; step 2: 98 °C for 10 seconds; step 3: 65 °C for 20 seconds; step 4: 72 °C for 130 seconds; step 2-4 were repeated 25 times; step 5: 72 °C for 2 minutes. KLD treatment and *E*.*coli* transformation were performed as described before, and later mini and maxi-prep, as detailed above. *Ct*UGGT-ΔTRXL1:Litmus28i plasmid DNA, 3 mL at 400 ng/μL were obtained. The insert was then linearised, cloned into pHLsec vector (by Gibson Assembly) as described above, and after mini- and then maxi-prep, 3 mL of *Ct*UGGT-ΔTRXL1:pHL-sec plasmid DNA at 500 ng/μL were obtained.

***Cloning of CtUGGT-ΔTRXL2***. The *Ct*UGGT-ΔTRXL2 construct lacks residues *Ct*UGGT 417-650. The deletion of the *Ct*UGGT TRXL2 domain was performed starting from the gene of *Ct*UGGT in Litmus28 as described before, using forward (Δ2_F: 5’-GCCCTATCAAGACGGAAC-3’) and reverse (Δ2_R: 5’-AAATCTCCGGGGCTCGTC-3’) primers. PCR protocol: step 1: 98 °C for 30 seconds; step 2: 98 °C for 10 seconds; step 3: 64 °C for 20 seconds; step 4: 72 °C for 180 seconds; step 2-5 were repeated 25 times; step 6: 72 °C for 2 minutes. KLD treatment and *E*.*coli* transformation were performed as described before, and later mini and maxi-prep, as detailed above. *Ct*UGGT-ΔTRXL2:Litmus28i plasmid DNA, 3 mL at 300 ng/μL were obtained. The insert was then linearised, cloned into pHLsec vector (by Gibson Assembly) as described above, and after mini- and then maxi-prep, 3 mL of *Ct*UGGT-ΔTRXL2:pHL-sec plasmid DNA at 300 ng/μL were obtained.

***Cloning of CtUGGT-ΔTRXL3***. The *Ct*UGGT-ΔTRXL3 construct lacks residues *Ct*UGGT 666-870. The deletion of the *Ct*UGGT TRXL3 domain was performed starting from the gene of *Ct*UGGT in Litmus28i as described before, using forward (Δ3_F: 5'-ATTTCGGATCTCCCACAG-3') and reverse (Δ3_R: 5'-GTTCTTGTCTTCGGGGAAAATG-3') primers properly designed. PCR protocol: step 1: 98 °C for 30 seconds; step 2: 98 °C for 10 seconds; step 3: 62 °C for 20 seconds; step 4: 72 °C for 130 seconds; step 2-4 were repeated 25 times; step 5: 72 °C for 2 minutes. KLD treatment and *E*.*coli* transformation was performed as described before, and later mini and maxi-prep, as detailed above. *Ct*UGGT-ΔTRXL3:Litmus28i plasmid DNA, 3 mL at 500 ng/μL were obtained. The insert was then linearised, cloned into pHLsec vector (by Gibson Assembly) as described above, and after mini- and then maxi-prep, 3 mL of *Ct*UGGT-ΔTRXL3:pHL-sec plasmid DNA at 800 ng/μL were obtained.

#### Protein expression and purification

All *Ct*UGGT mammalian expression plasmids were transfected into FreeStyle^TM^ 293-F Cells. Human epithelial kidney (HEK) FreeStyle^TM^ 293-F Cells (ThermoFisher Scientific) at 10^6^ cells/mL suspended in FreeStyle^TM^ 293 Expression Medium (ThermoFisher Scientific) were transfected using the FreeStyle^TM^ MAX 293 Expression System (ThermoFisher Scientific). For 50 mL culture, the manufacturer suggested protocol was used: 62.5 μL of FreeStyle^TM^ MAX Reagent (ThermoFisher Scientific) and 62.5 μg of plasmid DNA were each diluted separately into 1 mL of OptiPRO^TM^ SFM reduced serum medium (ThermoFisher Scientific), then mixed, incubated for 10 min at room temperature and finally added to the cell suspension. Transfected cells were left shaking in 500 mL Erlenmeyer flasks with 0.2 μm vent caps (Corning) shaking at 135 revolutions per min (rpm) in a 37 °C incubator kept at 8% CO_2_. All the DNA plasmids contained signal sequences to ensure protein secretion into the supernatant. The cells were harvested and supernatants were separated from cells by centrifugation for 15 minutes at 4 °C and 3,000g. Constructs were purified as follow. The supernatants were added of phosphate buffer saline (PBS) to get 1X to final volume, of imidazole to 5mM final concentration and the pH was adjusted to 7.4-7.6 by adding the needed volume of 2M NaOH solution. The supernatants were filtered by vacuum through a 0.22 μm filter. All protein constructs were purified using Immobilised Metal Affinity Chromatography (IMAC) with a 1 mL HisTrap HP Ni IMAC column (GE Healthcare) (unless otherwise specified) and then size exclusion chromatography (SEC) with a HiLoad Superdex 200 16/60 column (GE Healthcare), unless otherwise specified. A HisTrap HP Ni IMAC column (GE Healthcare) already equilibrated with binding buffer (1x PBS, 5 mM imidazole and pH adjusted to 7.4-7.6 with the needed volume of 2M NaOH solution) was loaded with the cells’ supernatant treated as described above. The IMAC column was washed with 20 column volumes (cV) of binding buffer and protein eluted with a linear gradient over 20 cV from 0 % to 100 % of elution buffer (1x PBS, 400-500 mM imidazole, pH adjusted to 7.4-7.6 with the needed volume of 2M NaOH solution) at 2 mL/min. Peak fractions were pooled and concentrated using a PES membrane centrifugal ultrafiltration device (Sartorius), with the appropriate MW cut-off, to a maximum 5 mL volume. This concentrated protein sample was then loaded to the HiLoad Superdex 200 16/60 SEC column (GE Healthcare) equilibrated against the appropriate SEC buffer (see below). Peak fractions were pooled and concentrated as before; protein concentration measured by loading 1.5 μL of sample on a NanoDrop 1000 spectrophotometer (Thermo Scientific). The calculated ϵ_280_ (from http://protcalc.sourceforge.net/) was then used to estimate the protein concentration. Protein aliquots were frozen in liquid N_2_ and stored at -80 °C. SDS-PAGE of SEC fractions was used to assess purity. All SEC chromatography was run at 1 mL/min flow rate on ÄKTA Pure (room temperature) or ÄKTA Start (4 °C) systems (GE Healthcare) unless otherwise specified. ***Ct*UGGT**_**Kif**_ was expressed in 300 mL (2x150 mL) of HEK293F cells, supplementing the media with 5 μM kifunensine (Cayman Chemical Company). Transfected cells were left shaking at 135 rpm in 0.5 L Erlenmeyer flasks with 0.2 μm vent caps, at 37 °C and 8% CO_2_ incubator, for 6 days. Size exclusion chromatography was performed with SEC buffer: 20 mM Na-HEPES pH 7.5, 150 mM NaCl. The final concentration of *Ct*UGGT_Kif_ (1 mL volume) was 7.24 mg/mL. ***Ct*UGGT**^**S180C/T742C**^ was expressed in 400 mL of HEK293F cells. Transfected cells were left shaking at 135 rpm in 0.5 L Erlenmeyer flasks with 0.2 μm vent caps, at 37 °C and 8% CO_2_ incubator, for 4 days. The IMAC step used a 5 mL HisTrap column. Eluted fractions were analysed using SDS-PAGE and the protein was detected in the flow-through, having apparently failed to bind to the IMAC column. We can only speculate that the His-tag is either proteolysed or sequestered to the surface of the mutant in the IMAC binding buffer conditions. The 660 mL of flow-through was re-filtered through a 1 μm filter, then a 0.45 μm filter, then a 0.22 μm filter. It was diluted to 1 L with H_2_O and the pH adjusted to 8.5 with 2M NaOH solution, and thereafter loaded onto a HiPrep Q HP 16/60 anion exchange column equilibrated in buffer A: K_2_HPO_4_/KH_2_PO_4_ 20 mM pH 8.5, flowing at 4 mL/min. The column turned pink - probably because the pH indicator from the HEK293F cells medium is anionic at pH 8.5. The column was washed with 250 mL of buffer A and the protein eluted using buffer B (buffer A supplemented with NaCl to a final concentration of 1 M), in three steps: (i) 3.5 cV of 25% buffer B; (ii) 3.5 cV of 50% buffer B; (iii) 3.5 cV of 100% buffer B; 15 mL fractions were collected. Protein containing fractions were pooled and the 30 mL sample concentrated to 5 mL, then exchanged to 20 mM MES pH 6.5, 50 mM NaCl in two 150 KDa MWCO spin concentrators. The 5 mL of *Ct*UGGT^S180C/T742C^ in 20 mM MES pH 6.5, 50 mM NaCl were injected onto a HiLoad Superdex 200 16/60 column equilibrated in the same buffer, and run at 1 mL/min, collecting 1.5 mL fractions. Protein containing fractions were pooled and concentrated to 6.28 mg/mL (V=800 μL). ***Ct*UGGT**^**G177C/A786C**^ was expressed in 200 mL of HEK293F cells. Transfected cells were left shaking at 135 rpm in 0.5 L Erlenmeyer flasks with 0.2 μm vent caps, at 37 °C and 8% CO_2_ incubator, for 4 days. The cells’ supernatant was processed as previously described and run on a 5 mL HisTrap HP column (GE Life Sciences). Fractions were pooled and concentrated with a centrifugal concentrator before loading on a HiLoad Superdex 200 16/60 column in SEC buffer: 20 mM HEPES pH 7.2, 120 mM NaCl. Eluted fractions were analysed by SDS-PAGE and concentrated as before. The final concentration of *Ct*UGGT^G177C/A786C^ construct (0.8 mL volume) was 7.91 mg/mL. ***Ct*UGGT-ΔTRXL1** was expressed in 200 mL of HEK293F cells, harvested after 4 days of incubation. The construct was purified as per above. The final concentration of *Ct*UGGT-ΔTRXL1 construct (0.1 mL volume) was 0.4 mg/mL. ***Ct*UGGT-ΔTRXL2** was expressed in 150 mL of HEK293F cells. Transfected cells were left shaking at 135 rpm in 0.5 L Erlenmeyer flasks with 0.2 μm vent caps, at 37 °C and 8% CO_2_ incubator, for 3 days. The protein was purified from the cells’ supernatant as described. IMAC elution fractions were pooled and concentrated and injected in two 5 mL batches onto the SEC column (GE Healthcare), with SEC buffer: 20 mM HEPES pH 7.2, 120 mM NaCl. Elution fractions containing the protein were then pooled based on SDS-PAGE analysis. ***Ct*UGGT-ΔTRXL3** was expressed in 200 mL of HEK293F cells, harvested after 4 days of incubation. The construct was purified as per above. The final concentration of *Ct*UGGT-ΔTRXL3 construct (0.2 mL volume) was 12.80 mg/mL.

#### Protein crystallisation and cryoprotection

***Ct*UGGT**_**Kif**_. A *Ct*UGGT_Kif_ crystal grew in a sitting drop from protein at 7.24 mg/mL, in condition 34 of the MORPHEUS screen (Molecular Dimensions, (Gorrec, 2009) [0.09 M NPS: 0.03 M Sodium nitrate, 0.03 M Sodium phosphate dibasic, 0.03M Ammonium sulphate; 0.1 M buffer System 3: Tris Bicine pH 8.5; 8.530% v/v; Precipitant Mix 2: 40% v/v Glycerol, 20% w/v PEG 4000] mixed in protein:mother liquor ratio 100 nL:100 nL. The crystal grew at 18 °C and it was flash-cooled in liquid N_2_. Details described in the Open Laboratory Notebook at https://doi.org/10.5281/zenodo.3608191. ***Ct*UGGT-ΔTRXL2**. A *Ct*UGGT-ΔTRXL2 crystal grew at 18 °C from protein concentrated to 6.5 mg/mL and mixed in 133:67 nL protein:mother liquor ratio with solution 2 of the JCSG+ crystallisation screen (Molecular Dimensions, (Newman et al., 2005)) in a sitting drop: 0.1 M sodium citrate pH 5.5, 20% w/v PEG 3,000. The crystal was cryo-protected with 20% glycerol in mother liquor and cryo-cooled with liquid nitrogen. ***Ct*UGGT**^**S180C/T742C**^. Crystal growth, cryoprotection and X-ray data collection are described in the Open Laboratory Notebook at https://doi.org/10.5281/zenodo.1345671. Briefly: the P3_2_12 *Ct*UGGT^S180C/T742C^ crystal grew from sitting drop from protein at 6.28 mg/mL in HEPES 20 mM pH 6.5, 50 mM NaCl, 5 mM UDP-Glc, 1 mM CaCl_2_ mixed in protein:mother liquor ratio 100 nL:100 nL with condition 57 of the MORPHEUS2 screen (Gorrec, 2015) [2 mM Lanthanides, 0.1 M buffer System 6 (1.0 M, pH 8.5 at 20 °C, Gly-Gly, AMPD), 36 % v/v Precipitant Mix 5 (30% w/v PEG 3000, 40% v/v 1, 2, 4-Butanetriol, 2% w/v NDSB 256)]. The crystal grew between day 57 and day 71, at 18 °C. The crystal was flash-cooled in liquid N_2_. The P2_1_2_1_2_1_ *Ct*UGGT^S180C/T742C^ crystal grew from mixing protein at OD_280_=7.29 in HEPES 20 mM pH 6.5, 50 mM NaCl, 5 mM UDP-Glc, 1 mM CaCl_2_ with condition 14 of the JCSG+ screen (0.2 M Sodium thiocyanate, 20% w/v PEG 3350) in protein:mother liquor ratio 133 nL:66 nL. The crystal grew in a sitting drop two days at 18 °C and broke into smaller pieces upon fishing. The crystal was cryoprotected in liquid N_2_ after quick transfer to a solution obtained by mixing 2 μL of ethylene glycol (EG) in 8 μL of mother liquor (i.e. 20% EG). ***Ct*UGGT**^**G177C/A786C**^: Initial *Ct*UGGT^G177C/A786C^ crystals grew from a solution of mother liquor: 16.54% w/v PEG 4,000, 0.03 M citric acid pH 5.3, 0.07 M citric acid pH 6.0, 12.75% v/v isopropanol. The crystals initially diffracted to 25 Å only and it was decided to dehydrate them by re-equilibrating the crystallization drop against a PEG 6,000-containing mother liquor reservoir: 13 μL of mother liquor were taken out of the 50 μL in the reservoir, replaced with 13 μL of a solution of 50% w/V PEG 6,000 in mother liquor, and the plate re-sealed. After undergoing dehydration for a week, one crystal was flash frozen in liquid N_2_ for data collection.

#### UGGT-mediated re-glucosylation of urea-misfolded bovine thyroglobulin (UDT)

Bovine thyroglobulin (Sigma-Aldrich) was denatured with urea following the protocol in (Trombetta et al., 1989), briefly here: 20 mg of thyroglobulin (TG) powder was dissolved in 1 ml of ‘Column buffer’ using a vortex mixer and spun at 12000xg for 1 min to ensure that mixing occurred. The thyroglobulin was then injected onto a SuperoseTM 6 10/300 Increase gel filtration column (GE Life Sciences) which had been equilibrated with the ‘Column buffer’. SDS-PAGE analysis of the elution profile was used to select fractions containing pure protein, which were pooled and spin-concentrated to 2 ml. The thyroglobulin was then dialysed against 200 ml of ‘Denaturation buffer’ at room temperature for 36 hours (with fresh buffer changes at 12, 70 and 24 h) to achieve a dialysis exchange factor of 1,000,000x. After 36 h, the thyroglobulin was dialysed against 200 ml of ‘Renaturation buffer’ for 96 h at 4 °C (with fresh buffer changes at 48, 60 and 84 h). The urea-treated thyroglobulin was diluted to 1 ml with ‘Column buffer’, spin-filtered and run down a SuperoseTM 6 10/300 gel filtration column equilibrated with ‘Column buffer’. After SDS-PAGE analysis, fractions containing pure protein were pooled and concentrated. The pooled fractions gave the UDT used in the activity assays. Each reaction mixture contained 100 μg of UDT, 86 μM UDP-Glucose, 8.6 mM CaCl_2_, 8.6 mM Tris-HCl pH 8.0 and 45 pmol of *Ct*UGGT enzyme. The reaction mixtures were set up at 37 °C. Each reaction was 70 μL to start with, in triplicate. 10 μL aliquots were taken at each time point (5’, 15’, 30’, 1 h, 2 h and O/N), and the re-glucosylation quenched by addition to each 10 μL aliquot of 1 μL of PNAGaseF denaturing buffer, then heating for 10 min at 90 °C. Then 5 μL of 10X PNGase glycobuffer 2 (NEB), 5 μL of NP40 10%, 1 μL of PNGase F (NEB) at 1 mg/mL and 27 μL of water were added to each sample for the overnight digestion with PNGase F. The *N*-linked glycan were labelled with anthranilic acid (2-AA) (Sigma-Aldrich), purified by adsorption to Speed-amide SPE columns and detected by normal-phase high-performance liquid chromatography, see (Caputo et al., 2016). The amount of re-glucosylation was measured in comparison to control by measuring the peak area of the PNGase F released 2-AA-labelled species Man_9_GlcNAc_2_ and Glc_1_Man_9_GlcNAc_2_ using Waters Empower software. This allows the % of glucosylation to be determined as the amount of Glc_1_-species (Peak Area Glc_1_Man_9_GlcNAc_2_) expressed as a fraction of the total of the two species (Peak Area of Glc_1_Man_9_GlcNAc_2_ + Man_9_GlcNAc_2_).

#### X-ray diffraction data collection and processing

***Ct*UGGT**_**Kif**_: diffraction data were collected on I04@DLS, at a wavelength λ=0.9763 Å, beam size 80x20μm, 0.2° oscillation. Batches 2,3: plate set at 2.9 Å max resolution; batches 4,5, plate set at 3.5 Å max resolution. Batch 2: 450 images, 0.10 s exposure, Transmission T=70%. Batches 3,4: 500 images, 0.20 s exposure, T=100%. Batch 5: 350 images, 0.50 s exposure, T=100%. Recentring followed after each exposure. ***Ct*UGGT-ΔTRXL2**: data were collected on I04@DLS, at a wavelength λ=0.97950 Å, beam size 43×30 μm, 0.15° oscillation, 1200 images, 0.02 s/image and T=100%; plate set at 4.5 Å max resolution. ***Ct*UGGT**^**S180C/T742C**^: data were collected on I24@DLS, at a wavelength λ=0.96860 Å, beam size 50×50 μm, 0.10° oscillation, 1800 images, 0.10 s/image and T=30%; plate set at 3.5 Å max resolution. ***Ct*UGGT**^**G177C/A786C**^: data were collected on I04@DLS, at wavelength λ=0.97949 Å, beam size 19×10 μm, 0.10° oscillation, 1800 images, 0.10 s/image and T=100%; plate set at 4.5 Å max resolution.

All datasets were processed with the autoPROC suite of programs (Vonrhein et al., 2011). Table S2 contains the data processing statistics.

#### Crystal structure determination and refinement

***Ct*UGGT**_**Kif**_ (PDB ID 6TRF): Phaser (McCoy et al., 2007) was run in all primitive orthorhombic space groups searching for one copy of PDB ID 5NV4 from which TRXL2 was removed (declaring a RMSD_Cα_ of 2.0 Å - Phaser refined it to 0.77 Å). The results were clearly best in P2_1_2_1_2_1_ (RF Z-score 7.0; TF Z-score: 10.4; Refined TFZ-equiv: 16.3; LLG: 114; Refined TF Z-score: 16.3, Refined LLG: 208. wR=0.58). The first map obtained in autoBUSTER (Blanc et al., 2004) from this MR model (which lacks TRXL2) showed strong density for the TRXL2 domain. The TRXL2 domain was added by superposing PDB ID 5NV4 onto the model, and real-space fitting the domain to the Fo-Fc map in CCP4-coot (Emsley et al., 2010). The structure was refined in autoBUSTER with one TLS body per domain with external restraints (Smart et al., 2012) to PDB ID 5NV4.

***Ct*UGGT-ΔTRXL2** (PDB ID 6TS2): Molecular replacement with the program CCP4-Molrep was initially attempted using *Ct*UGGT PDB entry 5NV4 with the TRXL2 domain removed. Electron density for the TRXL3 domain (residues 667-879) was poor. This suggested that upon deletion of TRXL2, the relative orientation of TRXL3 with respect to the rest of the protein was also changed. The TRXL3 domain was therefore also cut from the search model. Three copies of this model were placed with CCP4-Molrep. A first round of refinement was carried out in autoBUSTER with one TLS body per domain, and one rigid body per domain, with automated NCS restraints and external secondary structure restraints to the deposited 5NV4 structure (R=35.0%, Rfree=37.6%). The phases showed positive difference density in regions close to the loose ends of the search model on either side of TRXL3 for copies A, B, C, suggesting that indeed the deletion of TRXL2 caused TRXL3 to rearrange. Two copies of the TRXL3 domain were then placed with CCP4-Molrep, clearly belonging to two of the molecules in the asymmetric unit. An additional search for a third TRXL3 copy gave a convincing solution that did not appear to belong to the three molecules so far placed, highlighting the possible presence of a fourth copy in the asymmetric unit. This model comprising two copies of *Ct*UGGT-ΔTRXL2, a *Ct*UGGT-ΔTRXL2-ΔTRXL3 model and a TRXL3 domain for a fourth copy was subject to refinement with the same protocol as above (R=31.9% Rfree=33.3%). After this refinement, electron density for the missing TRXL3 domain and the remaining domains of the fourth copy of the molecule was visible in the map. One of the *Ct*UGGT-ΔTRXL2 molecules was superposed onto the fourth copy’s TRXL3 domain, followed by rigid body fitting of the bulk of the final copy in Coot (Emsley et al., 2010). The final model was refined in autoBUSTER with one set of TLS thermal motion tensors per domain and non-crystallographic symmetry and external restraints to the PDB ID 5NV4 structure.

***Ct*UGGT**^**S180C/T742C**^ (PDB ID 6TRT): CCP4-Molrep was run against the *Ct*UGGT^S180C/T742C^ data in P3_1_12 and P3_2_12 searching with a copy of PDB ID 5NV4 from which all three TRXL1,2,3 were removed (leaving only TRXL4, BS1,BS2 and GT24 domains). The results were clearly better in P3_2_12 (P3_2_12 has wR=0.606, Score=0.435, TF/sigma=10.35, Contrast=9.21 versus P3_1_12 wR=0.637, Score=0.372, TF/sigma=5.61, Contrast=4.76). The first electron density map obtained in autoBUSTER (Blanc et al., 2004) from this MR model shows strong density for the TRXL3 domain and the Ca^2+^ site in the GT24 domain. The TRXL3 domain was added by superposing PDB ID 5NV4 onto the model, and real-space fitting the domain to the Fo-Fc map in CCP4-coot (Emsley et al., 2010). After one more round of refinement, the TRXL1 domain was added in the same way. Finally, the TRXL2 domain was added by molecular replacement with CCP4-Molrep in presence of the rest of the structure. The structure was refined in autoBUSTER (Blanc et al., 2004) with one TLS body per domain, one rigid body per domain, with external restraints (Smart et al., 2012) to PDB ID 5NV4. Fo-Fc residuals on two sites (the catalytic site and a crystal contact between TRXL2 and one of its symmetry mates) suggested a lanthanide ion from the crystallisation mix (which contains Y^3+^, Tb^3+^, Er^3+^, Yb^3+^). The ions are likely either Er^3+^ or Tb^3+^, which are known to substitute for Ca^2+^ and Mn^2+^ in protein coordination sites (Kastritis et al., 2017). At the wavelength of data collection, λ=0.96861 Å, f'_Er3+_=-1.7235 e^-^ and f''_Er3+_=8.2682 e^-^, while f'_Tb3+_=-1.046 e^-^ and f''_Tb3+_=6.9753 e^-^. Peaks at +9.4 and +7.4 sigmas are indeed visible at these two sites in the anomalous Fourier difference map. The ions were modelled as Tb^3+^, with a Tb^3+^-O distance of 2.4 ± 0.3 Å (coordinating residues: site 1: D1302, D1304, D1435; site 2: E774 from a symmetry mate, E713, E716 and D818).

***Ct*UGGT**^**G177C/A786C**^ (PDB ID 6TS8): The *Ct*UGGT^G177C/A786C^ crystal structure was initially phased by Molecular Replacement with Molrep searching in space group P4_3_ for two copies of PDB ID 5NV4 from which TRXL1 and TRXL2 were removed. The first map obtained in autoBUSTER (Blanc et al., 2004) from this MR model showed density for the missing domains, which were added by superposing PDB ID 5NV4 onto the model, and real-space fitting the TRXL1 and TRXL2 domains to the Fo-Fc map in CCP4-coot (Emsley et al., 2010). The structure was refined in autoBUSTER with one TLS body per domain with external and automated NCS restraints (Smart et al., 2012) to PDB ID 5NV4. Portions of the catalytic domain are disordered in the crystal and could not be traced.

Table S3 reports the Rfactors and geometry statistics for all models after the final refinements.

#### Homology modelling of the *Drosophila melanogaster* (*Dm*UGGT) structure

Modeller (Webb and Sali, 2016) was used to homology model the *Dm*UGGT structure, after aligning the sequences of the *Dm*UGGT and *Ct*UGGT proteins with Clustal Omega (Sievers and Higgins, 2018), using PDB ID 5NV4 as a template, and enforcing the disulfide bonds *Dm*UGGT C109:C123, C1348:C1441, C1437:C1455.

#### Fitting of the *Td*UGGT:FAb structure in the negative stain EM map

The crystal structures of *Td*UGGT catalytic domain (PDB ID 5H18, residues 1190-1466) and *Td*UGGT N-terminal portion (PDB ID 5Y7O, residues 29-1042) were aligned with the full-length *Ct*UGGT intermediate structure (PDB ID 5MU1, residues 1190-1466) in Coot (Emsley et al., 2010). Modeller (Webb and Sali, 2016) was then used to complete the *Td*UGGT structure, homology modelling the missing portions 158-165; 251-282; 403-414; 684-693; 738-741; 756-759; 1038-1150; 1380-1384 (after aligning the sequences of the *Td*UGGT (http://genome.fungalgenomics.ca *Thermomyces dupontii* NRRL 2155 Talth1p4_002475) and CtUGGT proteins with Clustal Omega (Sievers and Higgins, 2018)). The disulfide bonds *Td*UGGT C141:C153, C1322:C1415, C1411:C1429 were enforced. The fits of *Td*UGGT and anti- *Td*UGGT Fab models to the negative stain EM map (EMDB accession number EMD-30386) and to its inverse hand were carried out in Chimera (Pettersen et al., 2004) . Both for original and inverse hand map, the *Td*UGGT homology model was first aligned manually with the map, low-pass filtered to a resolution of 25 Å, then fitted to the EM map using the Fit in map tool in Chimera. After fitting the *Td*UGGT model, a Fab model from PDB ID 1FGN was fitted to the map with the same Fit in map tool in Chimera, again after low-pass filtering the PDB model to 25 Å. Final real space CCs in the original and inverse hand maps: ^ori^CC_*Td*UGGT_=0.89; ^ori^CC_Fab_=0.90; ^inv^CC_*Td*UGGT_=0.90; ^inv^CC_Fab_=0.90. The two models were deposited in PDB-DEV, accession code PDBDEV_00000054.

#### Frequency distributions of distances from glycoprotein residues to N-linked glycosylation sites

Starting with a list of 1372 Uniprot entries annotated as human glycoprotein for which some structural information is available, we selected 1244 Protein Data Bank (PDB) entries that contain structure for more than 90% of the primary sequence and include structural information for at least one N-linked glycan. For each entry, the distances from every residue atom and atoms in N-glycosylated asparagine residues were computed, and the distances to the closest and second-closest glycans were histogrammed. As a control for glycoprotein size, we also histogrammed all aminoacid-aminoacid distances within each same structure. The same data were sorted numerically to produce cumulative distributions.

#### Computational simulations

**System Preparation**. We used as initial structures the four available *Ct*UGGT structures , which we call 'open' (PDB ID 5MZO); 'intermediate' (PDB ID 5MU1); 'closed' (PDB ID 5N2J); the mutant D611C-G1050C ‘closed-like’ (PDB ID 5NV4); and the newly determined ‘new-intermediate’ *Ct*UGGT_Kif_ (PDB ID 6TRF). Starting from each structure, we performed 250 ns all-atom Molecular Dynamics (MD) simulations using the AMBER force field and package (Maier et al., 2015) and analyzed the resulting dynamics and conformational landscape using Principal Component (PC) analysis. For each system, all non-protein molecules (carbohydrates and ions) were removed from the crystal structure. Gap regions (residues 242-273, 1152-1187 and 1334-1342) were completed and refined using Modeller (Eswar et al., 2008). Standard protonation states were assigned to titratable residues (Asp and Glu are negatively charged; Lys and Arg are positively charged). Histidine protonation was assigned favoring formation of hydrogen bonds in the crystal structure. The complete protonated systems were then solvated by a truncated cubic box of TIP3P waters, ensuring that the distance between the biomolecule surface and the box limit was at least 10 Å.

**MD simulations**. The systems were prepared with the tleap module from the AMBER package using the ff14SB/TIP3P force fields for amino acid/water molecules, respectively (Maier et al., 2015). Each system was first optimized using a conjugate gradient algorithm for 5000 steps, followed by 150 ps. Long constant volume MD equilibration, in which the first 100 ps were used to gradually raise the temperature of the system from 0 to 300 K (integration step = 0.0005 ps/step). The heating was followed by a 300 ps long constant temperature and constant pressure MD simulation to equilibrate the system density (integration step = 0.001 ps/step). During these temperature and density equilibration processes, the protein alpha-carbon atoms were constrained by 5 kcal/mol/Å force constant using a harmonic potential centered at each atom starting position. Next, a second equilibration MD of 500 ps was performed, in which the integration step was increased to 2 fs and the force constant for restrained alpha-carbons was decreased to 2 kcal/mol/Å, followed by 20 ns long MD simulation with no constraints. Finally, a 250 ns long MD simulation was carried out with no constraints and the 'Hydrogen Mass Repartition' method, which allows an integration step of 4 fs. The results of the latter step are described in this paper.

All simulations were performed using the pmemd.cuda algorithm from the AMBER package. Pressure and temperature were kept constant using the Monte-Carlo barostat and Langevin thermostat, respectively, using default coupling parameters. All simulations were performed with a 10 Å cutoff for nonbonded interactions, and periodic boundary conditions using the Particle Mesh Ewald summation method for long-range electrostatic interactions. The SHAKE algorithm was applied to all hydrogen-containing bonds in all simulations with an integration step equal or higher than 2 fs.

**PC calculations**. All trajectory processing and PC calculations were performed with the CPPTRAJ (Roe and Cheatham, 2013) module of the AMBER package. For each individual MD, PCs of the alpha-carbons were computed over an ensemble of 6000 trajectory frames representing the 250 ns long trajectories.

#### Mass spectroscopy: tryptic peptides

Protein samples were digested in-solution with sequencing grade trypsin (Promega). Briefly: samples were treated in 100 mM iodo-acetamide for 1 hour in dark to alkylate any free cysteines followed by denaturing with 8M urea for 40 min. The samples were further diluted with 50 mM ammonium bicarbonate to reduce the Urea concentration to 1 M. 1uL of 300ng/uL trypsin solution was added to each sample and incubated at 37 °C overnight. The resulting samples were directly analysed by LC-MS. Tryptic peptides of *Ct*UGGT, and the double mutants *Ct*UGGT^G177C/A786C^, *Ct*UGGT^G179C/T742C^ and *Ct*UGGT^S180C/T742C^ were separately run on an Dionex UltiMate3000 RSLC (Thermo Scientific) and electrosprayed directly into a Q Exactive mass spectrometer (Thermo Fischer Scientific) through an Flex ion-electrospray ion source (Thermo Fischer Scientific). Peptides were trapped on a C18 PepMap trapping column (μ-Precolumn, 300 μM I.D. x 5 mm, 100 μm particle size, 100 Å, Thermo Scientific) at a flow rate 10 μL/min. The trapping buffer was 0.05% v/v trifluoroacetic acid (TFA) in water (LC-MS grade). Samples were then separated using a C18, 75 μm x 25 cm (Acclaim PepMap nanoViper, part number 164941, 2.0 μm particle size, 100 Å, Thermo Scientific) analytical column (with mobile phases: 0.1% Formic acid in water (A) and 0.1% formic acid in acetonitrile (B)) at a flow rate of 300 nL/min, and the following gradients: minutes (mins) 0-5.5: 2% B; mins 5.5-10: 8% B; mins 10-40: 45% B; mins 40-41: 95% B; mins 41–46: 95% B; mins 46-60: 2% B.

Data were acquired in Data Dependent Mode (DDA) using the settings: chromatographic peak width: 20 s; resolution: 70,000; AGC target: 3x10^6^; maximum IT (injection time): 100 ms; scan range: 300 to 2000 m/z; ddMS2 resolution: 17,500; AGC target: 5x10^4^; maximum IT: 100 ms; loop count: 10 (*i*.*e*. Top 10); isolation width: 4.0 m/z; fixed first mass: 120.0 m/z. Data dependent (dd) settings: minimum AGC target: 5.0x10^3^; intensity threshold: 5.0 x10^4^; charge exclusion: 1; peptide match: preferred; exclude isotope: on; dynamic exclusion: 30.0 s. A normalized Collision energy (NCE) of 27 was used for the fragmentation of peptides in a high-energy collision dissociation (HCD) cell and the s-lens setting in the tune file was changed to 70.

*Data analysis* (*crosslinking and protein identification*): MassMatrix (version 2.4.2) was used for data analysis to find S-S cross linking and protein/peptide identification (Xu and Freitas, 2009). A customized database, containing the sequences of the proteins of interest, was used to perform searches. MS data were converted into .mgf format using MSconvert from the ProteoWizard toolbox (Chambers et al., 2012). Search parameters were as follows: maximum number of missed cleavages = 4; fixed modification = none; variable modifications: CAMC- Iodoacetamide derivative (Carbamidomethyl) of C and OxiM- Oxidation of M; disulphide bonds were considered as the crosslink (Cys-Cys, -2.02 Da); mass accuracy filter = 20 ppm for precursor ions; MS2 tolerance = 0.02 Da (values as per the MassMatrix user’s protocol) (Xu and Freitas, 2009). The quality of a peptide match is mainly evaluated by three statistical scores: pp, pp2, pptag. A peptide match with max (pp, pp2) > 2.7 and pptag > 1.3 is considered to be significant with p value < 0.05 (as documented in (Xu and Freitas, 2009)).

#### Structural analysis

Structural figures were made using PyMOL (Rigsby and Parker, 2016) and Chimera (Pettersen et al., 2004). Structural movies were made using VMD (Cross et al., 2009).

### Quantification and statistical analysis

X-ray diffraction data merging was carried out in autoPROC (Vonrhein et al., 2011) within the XDS (Kabsch, 2010) and Aimless (Evans and Murshudov, 2013) programs (default statistical analysis as per the references). The re-glucosylation experiments were carried out on n=3 independent samples per data point and the mean and standard deviation evaluated as documented in the user guide of GraphPad Prism, version 8.0.0 for Windows, GraphPad Software, San Diego, California USA, www.graphpad.com. When evaluating statistical significance of mass spectrometry data in MassMatrix, the quality of a peptide match is mainly evaluated by three statistical scores: pp, pp2, pptag. A peptide match with max (pp, pp2) > 2.7 and pptag > 1.3 is considered to be significant with p value <0.05 (Xu and Freitas, 2009).

## Supplemental information

Document S1. Figures S1–S4 and Tables S1–S4

Document S2. Article plus Supplemental Information
