# Supplementary material for: Clamping, bending, and twisting inter-domain motions in the misfold-recognizing portion of UDP-glucose: Glycoprotein glucosyltransferase
Source: Structure. 2021 Apr 1;29(4):357–370.e9. doi: 10.1016/j.str.2020.11.017 (PMC8024514; doi:10.1016/j.str.2020.11.017)
Supplement: Document S1. Figures S1–S4 and Tables S1–S4 [file mmc1.pdf]

Structure, Volume *29*

Supplemental Information

Clamping, bending, and twisting inter-domain motions in the misfold-recognizing portion of UDP-glucose: Glycoprotein glucosyltransferase

Carlos P. Modenutti, Juan I. Blanco Capurro, Roberta Ibba, Dominic S. Alonzi, Mauro N. Song, Snežana Vasiljević, Abhinav Kumar, Anu V. Chandran, Gabor Tax, Lucia Marti, Johan C. Hill, Andrea Lia, Mario Hensen, Thomas Waksman, Jonathan Rushton, Simone Rubichi, Angelo Santino, Marcelo A. Martí, Nicole Zitzmann, and Pietro Roversi

Supplementary Information Figures and Tables

Clamping, bending, and twisting inter-domain motions in the misfold-recognising portion of UDP-glucose:glycoprotein glucosyl-transferase.

Carlos P. Modenutti[1,2,¶], Juan I. Blanco Capurro[1,2,¶], Roberta Ibba[3,4,¶], Dominic S. Alonzi[3], Mauro N. Song[1,2], Snežana Vasiljević[3], Abhinav Kumar[3], Anu V. Chandran[3], Gabor Tax[5], Lucia Marti[6], Johan C. Hill[3], Andrea Lia[3,5,6], Mario Hensen[3], Thomas Waksman[3], Jonathan Rushton[3], Simone Rubichi[3,6], Angelo Santino[6], Marcelo A. Martí [1,2,*], Nicole Zitzmann [3,*] and Pietro Roversi [3,5,*].

1 Departamento de Química Biológica, Facultad de Ciencias Exactas y Naturales, Universidad de Buenos Aires, Ciudad Universitaria, Pab. II (CE1428EHA), Buenos Aires, Argentina

2 Instituto de Química Biológica de la Facultad de Ciencias Exactas y Naturales (IQUIBICEN) CONICET. Ciudad Universitaria, Pab. II (CE1428EHA), Buenos Aires, Argentina

3 Oxford Glycobiology Institute, Department of Biochemistry, University of Oxford, Oxford OX1 3QU, England, United Kingdom

4 Dipartimento di Chimica e Farmacia, Università degli Studi di Sassari, Via Muroni 23A, 07100 Sassari, SS, Italy

5 Leicester Institute of Structural and Chemical Biology, Department of Molecular and Cell Biology, University of Leicester, Henry Wellcome Building, Lancaster Road, Leicester, LE1 7RH, England, United Kingdom

6 Institute of Sciences of Food Production, C.N.R. Unit of Lecce, via Monteroni, I-73100 Lecce, Italy

¶ These authors contributed equally to this work

* Correspondence: marti.marcelo@gmail.com (M.A.M.), nicole.zitzmann@bioch.ox.ac.uk (N.Z.) and pr159@leicester.ac.uk (P.R.)

Supplementary Information Figures

Figure S1. Sequence conservation and HDX-MS deuterium uptake at the interface of the GT24 and βS1-βS2 domains, related to Figure 2A.

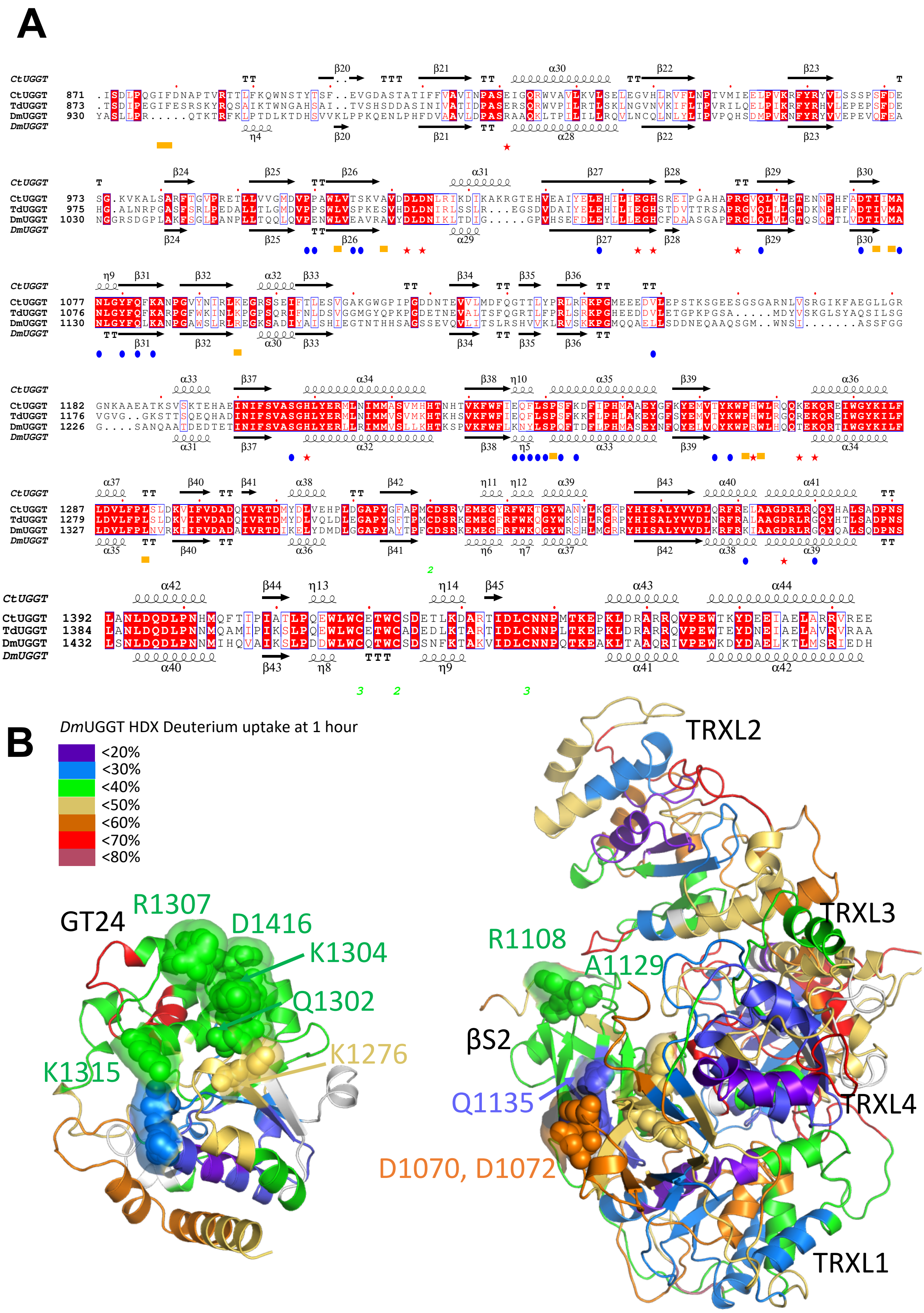

Figure S2. Fitting of full-length *Td*UGGT and Fab models in the negative-stain EM map for the complex of *Td*UGGT and an anti-*Td*UGGT Fab, related to Figure 2A.

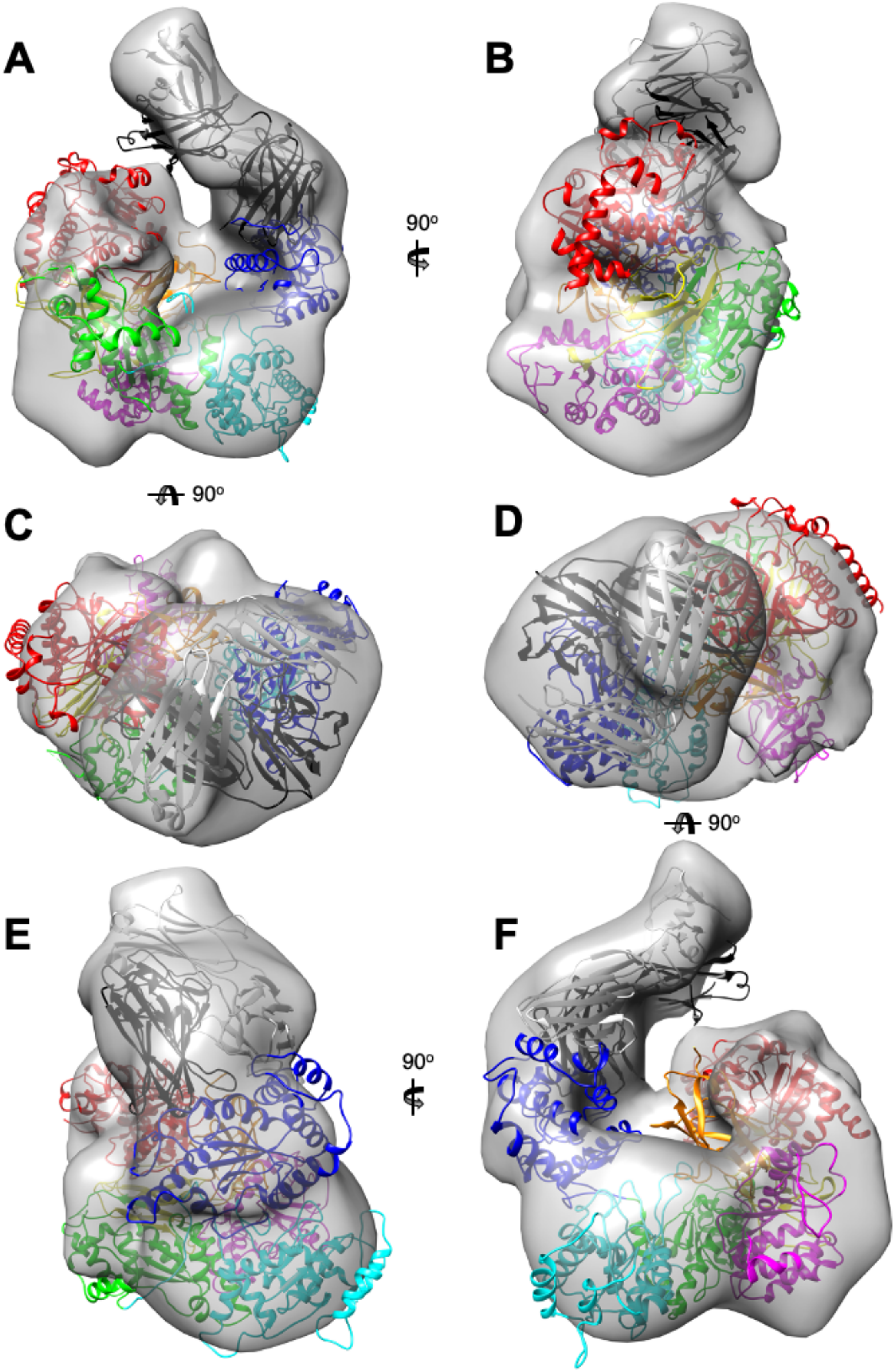

Figure S3. Projections of individual MD trajectories for *Ct*UGGT double Cysteine mutants onto the full conformational landscape of the wild type enzyme, coloured as a function of time, related to Figure 3.

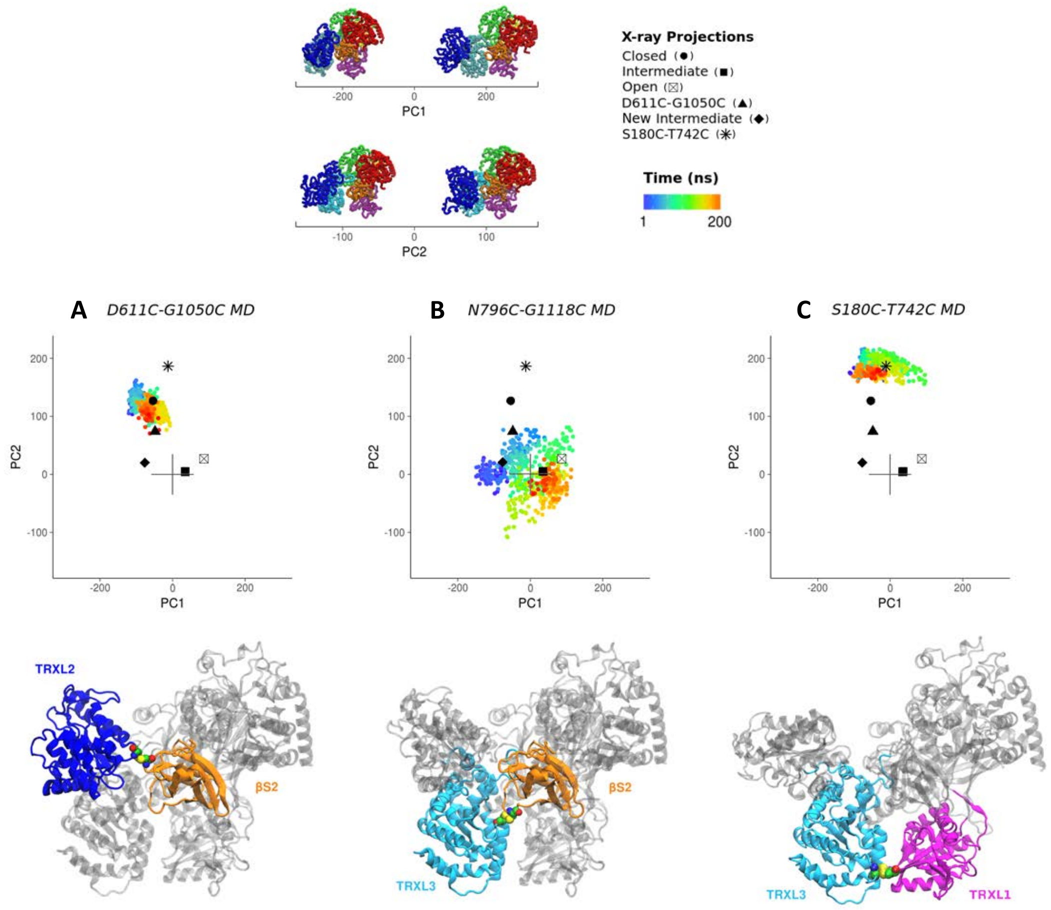

Figure S4. Mass spectrometry of tryptic peptides confirms the disulfides in the *Ct*UGGT double Cys mutants *Ct*UGGT$^{G177C/A786C}$, *Ct*UGGT$^{G179C/T742C}$ and *Ct*UGGT$^{S180C/T742C}$, related to Figure 6.

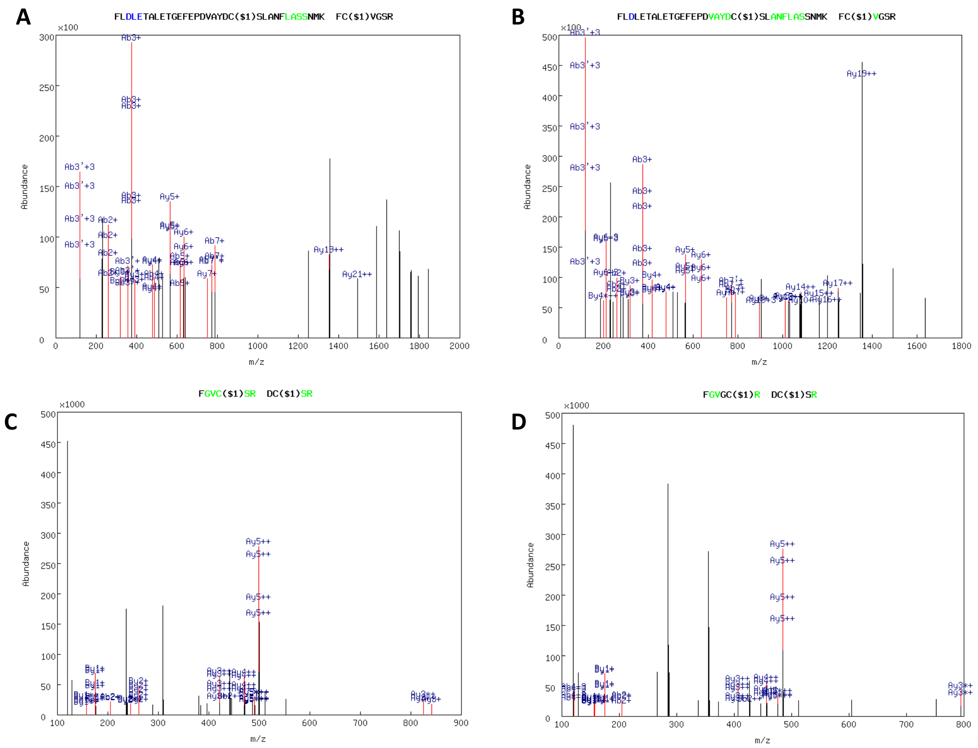

SI Appendix Tables.

Table S1. ***In vitro*** **UGGT substrates, related to Figure 4.**

List of various UGGT misfolded glycoprotein substrates described in the literature as UGGT substrates in *in vitro* experiments. No glycoproteins inferred to be UGGT substrates by *in cellula* experiments are included (see for example (Gardner and Kearse, 1999; Jin et al., 2007; Li et al., 2009; Pankow et al., 2015; Pearse et al., 2008)) nor glycoproteins that are *bona fide* UGGT substrates but whose structure has not been determined (Molinari et al., 2005; Trombetta et al., 1989)). (*): structures are available only for the pro-glycoprotein (previous to protease cleavage)

Substrate	PDB ID	Number of residues	Radius of Gyration (Å)	Reference
Crambe hispanica **crambin**	**1CRN**	**46**	**9.7**	(Dedola et al., 2014)
Hordeum vulgare **chymotrypsin inhibitor 2**	**2CI2**	**64**	**11.4**	(Caramelo et al., 2003)
**Human interleukin 8 (IL-8)**	**1ICW**	**72**	**12.5**	(Izumi et al., 2012)
**Human prosaposin A**	**5NXB**	**87**	**N/A(*)**	(Pearse et al., 2010)
**Bovine RNase BS-prot**	**2E33**	**104**	**14.7**	(Ritter and Helenius, 2000; Ritter et al., 2005)
**Bovine RNase B**	**2E33**	**124**	**14.3**	(Ritter et al., 2005; Ritter and Helenius, 2000; Sousa and Parodi, 1995)
Staphylococcus aureus **nuclease**	**1NUC**	**149**	**14.4**	(Ritter et al., 2005; Ritter and Helenius,

				2000; Sousa and Parodi, 1995)
***Trypanosoma cruzi* cruzipain**	**3I06**	**215**	**N/A(*)**	(Labriola et al., 1999)
**Soybean agglutinin**	**4D69**	**234**	**16.9**	(Keith et al., 2005)
**Human alpha-galactosidase**	**3HG5**	**390**	**21.5**	(Taylor et al., 2003)
**Human exo-(1,3)-β-glucanase**	**1H4P**	**407**	**20.3**	(Taylor et al., 2004)
**Human transferrin**	**6D04**	**678**	**29.7**	(Wada et al., 1997)

Table S2. *Ct*UGGT X-ray diffraction data collection statistics, related to STAR Methods section.

***Ct*UGGT Structure**	***Ct*UGGT$_{Kif}$**	**ΔTRXL2**	**G177C/A786C**	**S180C/T742C**
**PDB ID**	6TRF	6TS2	6TS8	6TRT
**Beamline, date**	I03@DLS, 01.05.2016	I04@DLS, 13.01.2018	I04@DLS, 08.10.2018	I24@DLS, 08.08.2018
**Space group (Z)**	$P2_12_12_1$ (4)	$P2_1$ (8)	$P4_3$ (8)	$P3_212$ (6)
**Wavelength (Å)**	0.97630	0.97950	0.97949	0.96861
**Cell dimensions *a, b, c* (Å)**	a=78.65, b=148.93, c=190.30	a=151.14 b=191.01 c=158.81	a=b=139.05 c=176.09	a=b=148.80 c=235.55
**α, β, γ (°)**	α=90.0, β=90.0, γ=90.0	α=90.0, β=117., γ=90.0	α=90.0, β=90.0, γ=90.0	α=90.0, β=90.0, γ=120.0
**Resolution range (Å)**	95.15-4.11 (4.49-4.11)	140.61-5.74 (6.15-5.74)	139.05-4.59 (5.32-4.59)	128.86-4.58 (5.13-4.58)
**R_{merge}**	0.149 (3.268)	0.150 (1.432)	0.275 (1.347)	0.118 (1.563)
**R_{meas}**	0.157 (3.376)	0.189 (2.146)	0.299 (1.453)	0.125 (1.650)
**$CC_{1/2}$**	0.997 (0.566)	0.994 (0.382)	0.991 (0.436)	0.995 (0.411)
**$I / \sigma(I)$**	9.9 (1.3)	3.6 (1.3)	5.5 (1.5)	10.6 (1.6)
**Completeness (%)**	90.2 (74.2)	91.5 (52.6)	87.8 (77.8)	90.1 (83.0)
**Redundancy**	10.9 (15.9)	5.6 (6.0)	6.7 (7.1)	8.9 (9.8)

Table S3. *Ct*UGGT crystal structures, refinement statistics, related to STAR Methods section.

Crystal form	*Ct*UGGT$_{Kif}$	ΔTRXL2	G177C/A786C	S180C/T742C
**PDB ID**	6TRF	6TS2	6TS8	6TRT
**Space group (Z)**	$P2_12_12_1$ (4)	$P2_1$ (8)	$P4_3$ (8)	$P3_212$ (6)
**Resolution (Å)**	95.1-4.1 (4.6-4.1)	140.6-5.7 (6.0-5.7)	139.0-4.6 (4.7-4.6)	128.9-4.6 (5.1-4.6)
**No. reflections**	7,503 (442)	17,358 (424)	5,723 (23)	9,528 (477)
R_{work} / R_{free}	0.25/0.31 (0.26/0.22)	0.17/0.24 (0.22/0.33)	0.20/0.23 (0.18/0.23)	0.29/0.30 (0.25/0.26)
**Atoms**	10,717	35,718	20,112	11,210
**<*B*-factor> (Å^2)**	270	134	173	143
**Rmsd$_{bonds}$(Å)**	0.006	0.009	0.01	0.006
**Rmsd$_{angles}$ (°)**	0.95	1.07	1.59	0.99
**Ramachandran outliers**	17/1309 (1.3%)	87/4431 (2.0%)	15/1285 (1.2%)	14/1363 (1.0%)
**Ramachandran allowed**	1292/1309 (98.7%)	4344/4431 (98.0%)	2392/2492 (96.0%)	1349/1363 (99.0%)
**Ramachandran favoured**	1235/1309 (94.3%)	3934/4431 (88.8%)	1977/2492 (79.3%)	1284/1363 (94.2%)

All structures were refined against X-ray data from one crystal only. Values in parentheses are for highest-resolution shell.

Table S4. Oligonucleotides , related to STAR Methods section.

Label	***Sequence***	Reference	Notes
CtUGGT_pHLsec_F primer	5'-GGTTGCGTAGCTGAAA CCGGTCAAGTCGCAGCCTCTCCA-3'	(Roversi et al., 2017)	Primer for Gibson Assembly
CtUGGT_pHLsec_R primer	5'-GATGGTGGTGCTTGGTACCC TCCCGAACCGTCTTGAC-3'	(Roversi et al., 2017)	Primer for Gibson Assembly
Δ1_F primer	5'-GAGTCTCTGTCCGTCAATGG-3'	This manuscript	Primer for mutagenesis to obtain CtUGGT_ΔTRXL1
Δ1_R primer	5'-AGAGGGGAAAGCGGCTTT-3'	This manuscript	Primer for mutagenesis to obtain CtUGGT_ΔTRXL1
Δ2_F primer	5'-GCCCTATCAAGACGGAAC-3'	This manuscript	Primer for mutagenesis to obtain CtUGGT_ΔTRXL2
Δ2_R primer	5'-AAATCTCCGGGGCTCGTC-3'	This manuscript	Primer for mutagenesis to obtain CtUGGT_ΔTRXL2
Δ3_F primer	5'-ATTTCGGATCTCCCACAG-3'	This manuscript	Primer for mutagenesis to obtain CtUGGT_ΔTRXL3
Δ3_R primer	5'-GTTCTTGTCTTCGGGGAAAATG-3'	This manuscript	Primer for mutagenesis to obtain CtUGGT_ΔTRXL3
G177C_F primer	5'-TCGGAAGTTTtgcGTTGGTTCCC-3'	This manuscript	Primer for mutagenesis to obtain CtUGGT^{G177C} mutation
G177C_R primer	5'-TCAAATGGCAGTGTCCGC-3'	This manuscript	Primer for mutagenesis to obtain CtUGGT^{G177C} mutation
V178C_F primer	5'-GAAGTTTGGCtgtGGTTCCCGTG-3'	This manuscript	Primer for mutagenesis to obtain CtUGGT^{V178C} mutation
V178C_R primer	5'-CGATCAAATGGCAGTGTC-3'	This manuscript	Primer for mutagenesis to obtain CtUGGT^{V178C} mutation
S180C_F primer	5'-TGGCGTTGGTtgcCGTGATGTGA-3'	This manuscript	Primer for mutagenesis to obtain CtUGGT^{S180C} mutation
S180C_R primer	5'-AACTTCCGATCAAATGGCAGTGTC-3'	This manuscript	Primer for mutagenesis to obtain CtUGGT^{S180C} mutation
T742C_F primer	5'-TCCCAAGGATtgcTCACGTTCCC-3'	This manuscript	Primer for mutagenesis to obtain CtUGGT^{T742C} mutation
T742C_R primer	5'-TTGTGGACAATGTCCAAC-3'	This manuscript	Primer for mutagenesis to obtain CtUGGT^{T742C} mutation
A786C_F primer	5'-CGCTTACGACtgtTCTCTAGCCAAC-3'	This manuscript	Primer for mutagenesis to obtain CtUGGT^{A786C} mutation
A786C_R primer	5'-ACATCTGGTTCGAACTCG-3'	This manuscript	Primer for mutagenesis to obtain CtUGGT^{A786C} mutation

Supplementary Information Figures Titles and Legends

Figure S1. Sequence conservation and HDX-MS deuterium uptake at the interface of the GT24 and βS1-βS2 domains, related to Figure 2A.

(A) The C-terminal parts of the sequences of *Ct*UGGT, *Td*UGGT and *Dm*UGGT (centred around the residues in the GT24:βS1-βS2 interface) are aligned and the conserved residues shown in white text over red squares. Similar residues are in red text over white squares with blue edges. Disulphide bonds are labelled in green under the Cys residues. The *Ct*UGGT (*Dm*UGGT) secondary structure is indicated above (below) its sequence. Blue dots: residues whose side chains are forming hydrogen bonds across the GT24:βS1-βS2 domains interface. Red stars: residues whose side chains are forming salt bridges across the GT24:βS1-βS2 domains interface. Orange squares: residues whose side chains are forming hydrophobic interactions across the GT24:βS1-βS2 domains interface. The sequences were aligned using Clustal Omega (Sievers and Higgins, 2018). The figure has been made using ESPript (Robert and Gouet, 2014);. **(B)** homology model of the *Drosophila melanogaster* UGGT (*Dm*UGGT), in cartoon representation; the GT24 domain has been split from the rest of the structure in order to expose the GT24:βS1-βS2 domains interface. The main residues in the same interface are in spheres and surface representation. The structure is coloured according to deuterium uptake at the 1 hour timepoint (Hydrogen Deuterium eXchange Mass Spectrometry (HDX-MS) data from (Calles-Garcia et al., 2017)), see legend in the inset.

Figure S2. Fitting of full-length *Td*UGGT and Fab models in the negative-stain EM map for the complex of *Td*UGGT and an anti-*Td*UGGT Fab, related to Figure 2A.

The homology model for *Td*UGGT (residues 29-1466) was coloured as follows: TRXL1 (residues 40-219): magenta; TRXL2 (residues 413-657): blue; TRXL3 (residues 658-898): cyan; TRXL4 (residues 239-412; 899-958): green; βS1 (residues 29-39; 220-238; 959-1037): yellow; βS2 (residues 1038-1151): orange; GT24 (residues 1190-1466): red. A generic Fab structure was chosen for the fitting of the anti-*Td*UGGT Fab antibody fragment (PDB ID 1FGN, 214+214 residues, MW=46927 Dalton), painted black (heavy chain) and white (light chain). The 25 Å negative-stain EM map is contoured at a contour level appropriate for enclosing the mass of the *Td*UGGT plus a Fab fragment (i.e. about 356,800 $Å^3$ corresponding to a mass of 295,000 Dalton, based on a specific volume of 1.21 Å per Dalton (Harpaz et al., 1994)). **(A), (B)** and **(C):** three views of the *Td*UGGT and Fab models fitted in the original hand of the negative stain EM map (with B and C rotated by 90˚ with

respect to view A around the centre of mass of the model, along the vertical and horizontal direction, respectively); **(D)**, **(E)** and **(F):** three views of the same *Td*UGGT and Fab models fitted to the inverse hand of the negative stain EM map (with E and F rotated by 90˚ with respect to view D around the centre of mass of the model, along the vertical and horizontal direction, respectively).

Figure S3. Projections of individual MD trajectories for *Ct*UGGT double Cysteine mutants onto the full conformational landscape of the wild type enzyme, coloured as a function of time, related to Figure 3.

Upper panels show the projections of individual MD trajectories onto the full conformational landscape as described by the first and second PCs, coloured as a function of time. Domains coloured as in Figure S1. Lower panels show the structure of each mutant, with the mutated cysteine residues drawn in sphere representation and domains containing the mutation shown in colour, with the rest of the protein is in grey. **(A)** MD trajectory projection starting from the crystal structure of the *Ct*UGGT$^{D611C/G1050C}$ mutant (PDB ID: 5NV4); **(B)** MD trajectory projection starting from the homology model of the *Ct*UGGT$^{N796C-G1118C}$ mutant, generated using the closed X-ray structure as template and Modeller.; **(C)** MD trajectory projection starting from the crystal structure of the *Ct*UGGT$^{S180C/T742C}$ mutant (PDB ID: 6TRT).

Figure S4. Mass spectrometry of tryptic peptides confirms the disulfides in the *Ct*UGGT double Cys mutants *Ct*UGGT$^{G177C/A786C}$, *Ct*UGGT$^{G179C/T742C}$ and *Ct*UGGT$^{S180C/T742C}$, related to Figure 6.

In peptide mass spectrometry, fragment ions that appear to extend from the amino- or carboxy-terminus of a peptide are termed "b" or "y" ions, respectively. **(A,B)** mass spectrometry detection of ions derived from fragmentation of the disulphide-bridged tryptic peptides ^{766}FLDLETALETGEFEPDVAYD**C**SLANFLASSNMK^{798} and ^{176}F**C**VGSR^{181} in the double mutant *Ct*UGGT$^{G177C/A786C}$. The ions confirm the establishment of the engineered disulphide bridge at positions 177-786 between the TRXL1 and TRXL3 domains. No peptides containing free Cys at either position 177 or 786 were detected; **(C)** mass spectrometry detection of ions derived from fragmentation of the disulphide-bridged tryptic peptides ^{741}D**C**SR^{744} and ^{176}FGV**C**SR^{181} in the double mutant *Ct*UGGT$^{G179C/T742C}$. The ions confirm the establishment of the engineered disulphide bridge at positions 179-742 between the TRXL1 and TRXL3 domains. No peptides containing free Cys at either position 179 or 742 were detected; **(D)** mass spectrometry detection of ions derived from fragmentation of the disulphide-bridged tryptic peptides ^{741}D**C**SR^{744} and ^{176}FGVG**C**RDVILYADITS^{191} in the double mutant *Ct*UGGT$^{S180C/T742C}$. The ions confirm the establishment of the engineered

disulphide bridge at positions 180-742 between the TRXL1 and TRXL3 domains. No peptides containing free Cys at either position 180 or 742 were detected.

References

Calles-Garcia, D., Yang, M., Soya, N., Melero, R., Ménade, M., Ito, Y., Vargas, J., Lukacs, G.L., Kollman, J.M., Kozlov, G., Gehring, K., 2017. Single-particle electron microscopy structure of UDP-glucose:glycoprotein glucosyltransferase suggests a selectivity mechanism for misfolded proteins. J. Biol. Chem. jbc.M117.789495. doi:10.1074/jbc.M117.789495

Caramelo, J.J., Castro, O.A., Alonso, L.G., De Prat-Gay, G., Parodi, A.J., 2003. UDP-Glc:glycoprotein glucosyltransferase recognizes structured and solvent accessible hydrophobic patches in molten globule-like folding intermediates. Proceedings of the National Academy of Sciences 100, 86–91. doi:10.1073/pnas.262661199

Dedola, S., Izumi, M., Makimura, Y., Seko, A., Kanamori, A., Sakono, M., Ito, Y., Kajihara, Y., 2014. Folding of Synthetic Homogeneous Glycoproteins in the Presence of a Glycoprotein Folding Sensor Enzyme. Angew. Chem. Int. Ed. 53, 2883–2887. doi:10.1002/anie.201309665

Gardner, T.G., Kearse, K.P., 1999. Modification of the T cell antigen receptor (TCR) complex by UDP-glucose:glycoprotein glucosyltransferase. TCR folding is finalized convergent with formation of alpha beta delta epsilon gamma epsilon complexes. Journal of Biological Chemistry 274, 14094–14099.

Harpaz, Y., Gerstein, M., Chothia, C., 1994. Volume changes on protein folding. Structure/Folding and Design 2, 641–649.

Izumi, M., Makimura, Y., Dedola, S., Seko, A., Kanamori, A., Sakono, M., Ito, Y., Kajihara, Y., 2012. Chemical synthesis of intentionally misfolded homogeneous glycoprotein: a unique approach for the study of glycoprotein quality control. J. Am. Chem. Soc. 134, 7238–7241. doi:10.1021/ja3013177

Jin, H., Yan, Z., Nam, K.H., Li, J., 2007. Allele-specific suppression of a defective brassinosteroid receptor reveals a physiological role of UGGT in ER quality control. Molecular Cell 26, 821–830. doi:10.1016/j.molcel.2007.05.015

Keith, N., Parodi, A.J., Caramelo, J.J., 2005. Glycoprotein tertiary and quaternary structures are monitored by the same quality control mechanism. Journal of Biological Chemistry 280, 18138–18141. doi:10.1074/jbc.M501710200

Labriola, C., Cazzulo, J.J., Parodi, A.J., 1999. Trypanosoma cruzi calreticulin is a lectin that binds monoglucosylated oligosaccharides but not protein moieties of glycoproteins. Mol. Biol. Cell 10, 1381–1394.

Li, J., Zhao-Hui, C., Batoux, M., Nekrasov, V., Roux, M., Chinchilla, D., Zipfel, C., Jones, J.D.G., 2009. Specific ER quality control components required for biogenesis of the plant innate immune receptor EFR. Proc. Natl. Acad. Sci. U.S.A. 106, 15973–15978. doi:10.1073/pnas.0905532106

Molinari, M., Galli, C., Vanoni, O., Arnold, S.M., Kaufman, R.J., 2005. Persistent glycoprotein misfolding activates the glucosidase II/UGT1-driven calnexin cycle to delay aggregation and loss of folding competence. Molecular Cell 20, 503–512. doi:10.1016/j.molcel.2005.09.027

Pankow, S., Bamberger, C., Calzolari, D., Martínez-Bartolomé, S., Lavallée-Adam, M., Balch, W.E., Yates, J.R., 2015. ∆F508 CFTR interactome remodelling promotes rescue of cystic fibrosis. Nature 528, 510–516. doi:10.1038/nature15729

Pearse, B.R., Gabriel, L., Wang, N., Hebert, D.N., 2008. A cell-based reglucosylation assay demonstrates the role of GT1 in the quality control of a maturing glycoprotein. J. Cell Biol. 181, 309–320. doi:10.1083/jcb.200712068

Pearse, B.R., Tamura, T., Sunryd, J.C., Grabowski, G.A., Kaufman, R.J., Hebert, D.N., 2010. The role of UDP-Glc:glycoprotein glucosyltransferase 1 in the maturation of an obligate substrate prosaposin. J. Cell Biol. 189, 829–841. doi:10.1083/jcb.200912105
Ritter, C., Helenius, A., 2000. Recognition of local glycoprotein misfolding by the ER folding sensor UDP-glucose:glycoprotein glucosyltransferase. Nat. Struct. Biol. 7, 278–280. doi:10.1038/74035
Ritter, C., Quirin, K., Kowarik, M., Helenius, A., 2005. Minor folding defects trigger local modification of glycoproteins by the ER folding sensor GT. The EMBO Journal 24, 1730–1738. doi:10.1038/sj.emboj.7600645
Robert, X., Gouet, P., 2014. Deciphering key features in protein structures with the new ENDscript server. Nucleic Acids Res. 42, W320–4. doi:10.1093/nar/gku316
Roversi, P., Marti, L., Caputo, A.T., Alonzi, D.S., Hill, J.C., Dent, K.C., Kumar, A., Levasseur, M.D., Lia, A., Waksman, T., Basu, S., Soto Albrecht, Y., Qian, K., McIvor, J.P., Lipp, C.B., Siliqi, D., Vasiljević, S., Mohammed, S., Lukacik, P., Walsh, M.A., Santino, A., Zitzmann, N., 2017. Interdomain conformational flexibility underpins the activity of UGGT, the eukaryotic glycoprotein secretion checkpoint. Proc. Natl. Acad. Sci. U.S.A. 114, 8544–8549. doi:10.1073/pnas.1703682114
Sievers, F., Higgins, D.G., 2018. Clustal Omega for making accurate alignments of many protein sequences. Protein Sci. 27, 135–145. doi:10.1002/pro.3290
Sousa, M., Parodi, A.J., 1995. The molecular basis for the recognition of misfolded glycoproteins by the UDP-Glc:glycoprotein glucosyltransferase. The EMBO Journal 14, 4196–4203.
Taylor, S.C., Ferguson, A.D., Bergeron, J.J.M., Thomas, D.Y., 2004. The ER protein folding sensor UDP-glucose glycoprotein–glucosyltransferase modifies substrates distant to local changes in glycoprotein conformation. Nat Struct Mol Biol 11, 128–134. doi:10.1038/nsmb715
Taylor, S.C., Thibault, P., Tessier, D.C., Bergeron, J.J.M., Thomas, D.Y., 2003. Glycopeptide specificity of the secretory protein folding sensor UDP-glucose glycoprotein:glucosyltransferase. EMBO reports 4, 405–411. doi:10.1038/sj.embor.embor797
Trombetta, S.E., Bosch, M., Parodi, A.J., 1989. Glucosylation of glycoproteins by mammalian, plant, fungal, and trypanosomatid protozoa microsomal membranes. Biochemistry 28, 8108–8116.
Wada, I., Kai, M., Imai, S., Sakane, F., Kanoh, H., 1997. Promotion of transferrin folding by cyclic interactions with calnexin and calreticulin. The EMBO Journal 16, 5420–5432. doi:10.1093/emboj/16.17.5420
